# Acupuncture for carpal tunnel syndrome: A systematic review and meta-analysis of randomized controlled trials

**DOI:** 10.3389/fnins.2023.1097455

**Published:** 2023-02-23

**Authors:** Qinjian Dong, Xiaoyan Li, Ping Yuan, Guo Chen, Jianfeng Li, Jun Deng, Fan Wu, Yongqiu Yang, Hui Fu, Rongjiang Jin

**Affiliations:** ^1^Yilong County Hospital of Traditional Chinese Medicine, Nanchong, Sichuan, China; ^2^Department of Rehabilitation Medicine, West China Second University Hospital, Sichuan University, Chengdu, China; ^3^School of Health Preservation and Rehabilitation, Chengdu University of Traditional Chinese Medicine, Chengdu, China; ^4^Integrated Traditional Chinese and Western Medicine Hospital of Panzhihua City, Panzhihua, China

**Keywords:** acupuncture, carpal tunnel syndrome, systematic review, meta-analysis, randomized controlled trial

## Abstract

**Background:**

The evidence for the effectiveness of acupuncture for patients with carpal tunnel syndrome (CTS) is insufficient. Therefore, this systematic review and meta-analysis aimed to evaluate the effectiveness of acupuncture on CTS through a comprehensive literature search.

**Methods:**

English and Chinese databases were searched from their inceptions until 27 October 2022 to collect randomized controlled trials (RCTs) that investigated the effect of acupuncture on CTS. Two reviewers independently selected studies that met the eligibility criteria, extracted the required data, assessed the risk of bias using version 2 of the Cochrane risk-of-bias tool for randomized trials (ROB 2), and evaluated the quality of reporting for acupuncture interventions using the Revised Standards for Reporting Interventions in Clinical Trials of Acupuncture (STRICTA). The primary outcomes were symptom severity and functional status, while secondary outcomes included pain intensity, responder rate, and electrophysiological parameters. Review Manager software (version 5.4.1) was used for data analysis. The certainty of the evidence was rated with GRADEpro (version 3.6) software.

**Results:**

We included 16 RCTs with a total of 1,025 subjects. The overall risk of bias was rated as low in one RCT, some concerns in 14, and high in one. Compared with night splints, acupuncture alone was more effective in relieving pain, but there were no differences in symptom severity and functional status. Acupuncture alone had no advantage over medicine in improving symptom severity and electrophysiological parameters. As an adjunctive treatment, acupuncture might benefit CTS in terms of symptom severity, functional status, pain intensity, and electrophysiological parameters, and it was superior to medicine in improving the above outcomes. Few acupuncture-related adverse events were reported. The above evidence had a low or very low degree of certainty.

**Conclusion:**

Acupuncture as an adjunctive treatment may be effective for patients with CTS. Additionally, more rigorous studies with objective outcomes are needed to investigate the effect of acupuncture in contrast with sham acupuncture or other active treatments.

**Systematic review registration:**

https://www.crd.york.ac.uk/PROSPERO/display_record.php?RecordID=329925, identifier CRD42022329925.

## 1. Introduction

Carpal tunnel syndrome (CTS), the common peripheral nerve entrapment syndrome, is caused by compression of the median nerve at the level of the wrist. The prevalence of CTS is 1–5% in the general population (Atroshi et al., 1999) and 7–10% in the working-age population (Spahn et al., 2012b; Feng et al., 2021). CTS can occur in one or both hands and is characterized by pain, numbness, and tingling in the median nerve distribution. In advanced cases, muscle atrophy may develop (Wipperman and Goerl, 2016). Being female, being obese, having to overuse the wrists, those who are pregnant, and those who are in perimenopausal age pose a greater risk of being affected by CTS (Spahn et al., 2012a; Graham et al., 2016). Patients with CTS frequently awaken from sleep due to worsening symptoms and have a lower quality of life. In addition, CTS is associated with reduced work time, decreased productivity, and disability (Daniell et al., 2009). Patients with CTS miss an average of 27 days of work per year, and the costs of CTS are estimated to exceed $2 billion annually in the United States (Palmer and Hanrahan, 1995).

Treatment strategies for CTS include non-surgical and surgical approaches. Given the invasive nature of the surgery, patients with CTS prefer to choose non-surgical management as an initial treatment (Shi and MacDermid, 2011; Calandruccio and Thompson, 2018). According to the American Academy of Orthopaedic Surgeons (AAOS) (Graham et al., 2016), there are various non-surgical treatments for CTS, such as immobilization (brace/splint/orthosis), steroid injections, and oral steroids. However, the evidence for the effectiveness of these non-surgical approaches is insufficient (Page et al., 2012; Padua et al., 2016). Moreover, certain undesirable adverse reactions limit the usage of treatments, such as splints and braces, which may influence sleep when used nightly (Manente et al., 2001), and steroid injections, which can lead to skin thinning, changes in pigmentation, and other adverse reactions (Chesterton et al., 2018). Therefore, it is necessary to explore effective and safe non-surgical interventions for patients with CTS.

Acupuncture is gaining popularity and acceptance worldwide and is widely used in neuro-musculoskeletal disorders (Qiao et al., 2020). Randomized controlled trials (RCTs) have investigated the effect of acupuncture as a monotherapy or adjuvant intervention on CTS, but their findings have been inconsistent. Previous systematic reviews of acupuncture for CTS were conducted by Sim et al. (2011) (6 RCTs), Choi et al. (2018) (12 RCTs), and Wu et al. (2020) (10 RCTs), and these systematic reviews suggested that there was not sufficient and convincing evidence to support the effectiveness of acupuncture on CTS. To further investigate this, we updated the systematic review and meta-analysis to include more objective outcomes and recent RCTs.

## 2. Methods and analysis

### 2.1. Study registration

We registered this systematic review and meta-analysis at PROSPERO: https://www.crd.york.ac.uk/PROSPERO/display_record.php?RecordID=329925 (Registration ID: CRD42022329925). This systematic review and meta-analysis was conducted according to A Measurement Tool to Assess Systematic Reviews (AMSTAR 2) (Shea et al., 2017) and reported in light of the Preferred Reporting Items for Systematic Review and Meta-Analysis (PRISMA) 2020 statement (Page et al., 2021).

### 2.2. Inclusion criteria

We included studies that met all of the following criteria:

#### 2.2.1. Type of studies

Our systematic review and meta-analysis included RCTs that evaluated the effectiveness of acupuncture in treating CTS and were published in either English or Chinese.

#### 2.2.2. Type of participants

Our study included adult patients (≥18 years old) with CTS diagnosed using electrophysiological assessment (e.g., nerve conduction studies) and/or a combination of symptoms history and physical examination (as per Erickson et al., 2019). There were no limitations on gender, ethnicity, severity, or duration of CTS among the study participants.

#### 2.2.3. Types of interventions

Experimental group: acupuncture alone or acupuncture plus other treatment(s) (e.g., wrist splinting, drugs, corticosteroid injection, and other non-traditional Chinese medicine). There were no restrictions on the types of acupuncture.

Control group: no treatment, sham acupuncture alone, other treatment, or sham acupuncture combined with other treatment(s).

Presence of cointerventions: cointerventions were required to be equal between the experimental and control groups.

#### 2.2.4. Types of outcomes

##### 2.2.4.1. Primary outcomes

Primary outcomes were symptom severity and functional status. Symptom severity was measured using the Boston Carpal Tunnel Questionnaire’s symptom severity scale (CTQ-SSS) and the global symptoms score (GSS), while functional status was assessed with the CTQ’s functional status scale (CTQ-FSS) and the disabilities of the arm, shoulder, and hand questionnaire (DASH).

##### 2.2.4.2. Secondary outcomes

Secondary outcomes included the following:

(1) Pain intensity: the visual analog scale (VAS) or the numerical rating scale (NRS);
(2) Electrophysiological parameters: compound muscle action potential (CMAP), sensory nerve action potential (SNAP), distal motor latency (DML), distal sensory latency (DSL), motor nerve conduction velocity (MNCV), and sensory nerve conduction velocity (SNCV);
(3) Responder rate: responder (symptom improved or greatly improved) and non-responder (symptom did not change or worsened); and
(4) Adverse events.

### 2.3. Exclusion criteria

Studies were excluded if they met one of the following conditions:

(1) Studies including patients with CTS from a special population, such as those with diabetes, who were pregnant, and those with rheumatoid arthritis;
(2) Patients who had surgery for CTS;
(3) Experimental and/or control group included other interventions of traditional Chinese medicine (e.g., Tuina and Chinese herbs);
(4) Studies that provided no details of control intervention;
(5) Studies with duplicate data; and
(6) If full texts were unavailable through all practical approaches.

### 2.4. Search strategy

The following databases were searched from their inceptions until 27 October 2022: PubMed, EMbase, the Cochrane Library, the Chinese Biomedical Literature Database (CBM), the China National Knowledge Infrastructure (CNKI), the Chinese Science and Technology Periodical Database (VIP), and the Wanfang database (Wanfang Data). We utilized Medical Subject Headings (MESH) and free terms related to acupuncture and CTS to build search strategies. The search strategies for the above databases are provided in Supplementary material 1. We manually searched gray literature, reference lists of relevant reviews, and trial registers (ClinicalTrials.gov and the Chinese Clinical Trials Registry). Meanwhile, relevant experts were consulted for potentially eligible studies.

### 2.5. Study selection

EndNote X9 was used to manage the literature. Two independent reviewers (PY and GC) conducted the study selection. After removing duplicates, irrelevant records which were screened according to titles or abstracts were excluded. Then, the rest records with full text were scrutinized to identify eligible studies. The two reviewers cross-checked their identified studies and discussed any disputes.

### 2.6. Data extraction

The data on the following aspects were extracted by two reviewers (JL and JD) independently:

(1) Study’s information: first author, year of publication, country, sample size, and information related to the risk of bias (e.g., randomization and blinding);
(2) Participants’ (study level) characteristics: age, gender, diagnostic criteria, duration, and severity of CTS;
(3) Experimental group’s details: protocol of acupuncture (type, acupoint selection, frequency, duration, etc.) and/or other cointervention(s) (type, frequency, duration, etc.);
(4) Control group’s details: protocol of comparators and/or other cointervention(s) (type, frequency, duration, etc.); and
(5) Outcomes’ information: primary and secondary outcomes, adverse events.

If there are multiple-arm RCTs, we included only data from the arms with interventions relevant to this study. Two reviewers cross-checked the extracted information. Any discrepancy was resolved through discussion. The authors would be contacted if there was missing information.

### 2.7. Assessment of risk of bias

Two independent reviewers (QD and XL) assessed the risk of bias in the included studies using version 2 of the Cochrane risk-of-bias tool for randomized trials (ROB 2). According to ROB 2, five domains of bias were evaluated: the randomization process, deviations from intended interventions, missing outcome data, measurement of the outcome, and selection of the reported result. Each domain of individual study and all included studies were rated as “low risk,” “some concerns,” or “high risk.” Any disagreements were resolved with a third reviewer (RJ).

### 2.8. Assessment of the reporting quality of the intervention

Two independent reviewers (HF and QD) utilized the Revised Standards for Reporting Interventions in Clinical Trials of Acupuncture (STRICTA) to evaluate the reporting quality of interventions for each included study based on the following six items (17 sub-items) (MacPherson et al., 2010): acupuncture rationale, details of needling, treatment regimen, other components of treatment, practitioner background, and control or comparator interventions. The third reviewer (RJ) participated in resolution of discrepancies.

### 2.9. Certainty of evidence assessment

Two independent reviewers (FW and YY) assessed the certainty of the evidence with the GRADE (Grading of Recommendations Assessment, Development, and Evaluation) system. Each outcome was assessed based on five aspects: limitations, inconsistency, indirectness, imprecision, and publication bias, and categorized as high, moderate, low, or very low evidential certainty. GRADEpro (Version 3.6) software was used to evaluate the evidence and summarize the findings.

### 2.10. Data analysis

We evaluated acupuncture’s effects as monotherapy and adjunctive treatment, respectively. If feasible, meta-analyses were conducted using post-intervention data when clinical homogeneity existed between studies. We calculated the mean difference (MD) with 95% confidence intervals (CIs) for continuous data measured by uniform standards. Otherwise, standardized mean differences (SMDs) and 95% CIs were evaluated. For dichotomous data (e.g., responder rate), we calculated the risk ratios (RRs) and 95% CIs. The Chi-square test with a significance level of *P* < 0.10 and *I*^2^ statistic were used to detect and quantify heterogeneity, respectively. The random-effects model (REM) was applied in meta-analyses if there was substantial heterogeneity (*P* < 0.1 or *I*^2^ value >50%). Otherwise, the fixed-effects model (FEM) was used. We conducted descriptive analyses when meta-analyses were not appropriate or possible. Review Manager software (version 5.4.1) was used for data synthesis.

## 3. Results

### 3.1. Study inclusion and characteristics

**FIGURE 1:**
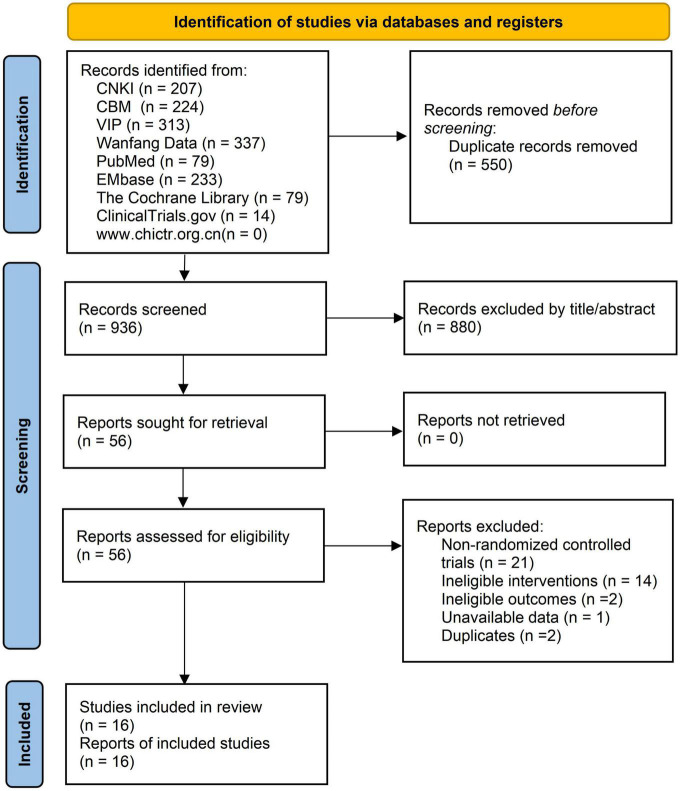
PRISMA flow diagram.

We obtained a total of 1,486 records in the literature search. After removing 550 duplicates, we excluded 880 irrelevant records based on their title and abstract. The full text of 56 remaining records was then evaluated, and 16 eligible studies (Kumnerddee and Kaewtong, 2010; Jin and Lang, 2011; Li, 2011; Yang et al., 2011; Yao et al., 2012; Ramin, 2013; Xiang et al., 2014; Hadianfard et al., 2015; Chung et al., 2016; Maeda et al., 2017; Ural and Özturk, 2017; Xie et al., 2018; Tezel et al., 2019; Bahrami-Taghanaki et al., 2020; Xiong, 2020; Huang and Lin, 2022) were included in the final analysis. A list of excluded records with reasons is provided in Supplementary material 2. The PRISMA flow chart presents the selection procedure (Figure 1). Of the included studies, eight were conducted in China (Jin and Lang, 2011; Li, 2011; Yang et al., 2011; Xiang et al., 2014; Chung et al., 2016; Xie et al., 2018; Xiong, 2020; Huang and Lin, 2022), two in the USA (Yao et al., 2012; Maeda et al., 2017), three in Iran (Ramin, 2013; Hadianfard et al., 2015; Bahrami-Taghanaki et al., 2020), two in Turkey (Ural and Özturk, 2017; Tezel et al., 2019), and one in Thailand (Kumnerddee and Kaewtong, 2010). The sample size of the studies ranged from 27 to 181, with a total of 1,025 participants. The mean age of participants varied between 36.4 and 53.6 years. Fifteen studies included patients with mild to moderate CTS, and one study (Jin and Lang, 2011) did not specify the severity of CTS. Five studies used acupuncture as monotherapy, while 11 studies investigated its adjunctive effect. Table 1 show the characteristics of the included studies.

**TABLE 1:** Characteristics of included studies.

References	Sample size (randomized/ analyzed)	Number of patient (randomized/ analyzed)	Age (E/C)	Gender (F/M)	Duration of CTS	Intervention			Control			Outcomes
						**Type**	**Frequency**	**Duration**	**Type**	**Frequency**	**Duration**	
Kumnerddee and Kaewtong, 2010	61/60	E: 30/30 C: 31/30	E: 50.37 ± 9.01 C: 51.73 ± 8.92	E: 26/4 C: 28/2	E: 12.12 ± 15.71 m C: 8.32 ± 7.68 m	EA	30 min/session 2 sessions/week	5 weeks	Night splints	Every night	5 weeks	CTQ-SSS CTQ-FSS VAS
Jin and Lang, 2011	50/50	E: 25/25 C: 25/25	E: 44 ± 6 C: 44 ± 4	E: 14/11 C: 12/13	E: 3.51 ± 0.5 m C: 3.65 ± 1.5 m	EA	30 min/session 1 session/day	20 days	Mecobalamin tablets	0.5 mg/time, tid	20 days	CAMP DML SNCV Responder rate
Li, 2011	80/80	E: 40/40 C: 40/40	E: 42.25 ± 9.73 C: 41.03 ± 10.07	E: 29/11 C: 31/9	NI	EA + medicine	40 min/session 5 sessions/week	4 weeks	Medicine (diclofenac sodium + mecobalamin tablets + vitamin B1 + vitamin B6 + dibazol tablets)	Diclofenac sodium: 25 mg, tid; Mecobalamin tablets: 500 μg, tid; Vitamin B1: 20 mg, tid; Vitamin B6: 20 mg, tid; Dibazol tablets: 10 mg, tid.	Diclofenac Sodium: 2 weeks; Mecobalamin/ vitamin B1/vitamin B6/dibazol tablets: 4 weeks	CTQ-SSS CTQ-FSS CMAP SNAP SNCV DML
Yang et al., 2011	77/77	E: 38/38 C: 39/39	E: 49.3 ± 8.9 C: 49.9 ± 10.3	E: 32/6 C: 30/9	E: 7.6 ± 3.8 m C: 7.7 ± 3.2 m	MA	30 min/session 2 sessions/week	4 weeks	Prednisolone	1–2 weeks: 20 mg daily; 3–4 weeks: 10 mg daily	4 weeks	GSS CMAP SNAP MNCV SNCV DML DSL
Yao et al., 2012	41/41	E: 21/21 C: 20/20	E: 53.6 ± 7.65 C: 48.5 ± 10.5	E: 14/7 C: 16/4	E: 74.4 ± 65.4 m C: 49.6 ± 53.7 m	MA + night splints	20 min/session 1 session/week	6 weeks	Sham acupuncture + night splints	Sham acupuncture: 20 min/session 1 session/week; Night splints: every night	6 weeks	CTQ-SSS CTQ-FSS
Ramin, 2013	52/52	E: 26/26 C: 26/26	47.61 ± 11.53	46/6	4.02 ± 4.84 m	EA	30 min/session 3 sessions/week	4 weeks	Prednisolone	5 mg daily	4 weeks	CMAP SNAP MNCV SNCV DML DSL
Xiang et al., 2014	60/60	E: 30/30 C: 30/30	E: 45.78 ± 9.05 C: 46.02 ± 8.93	E: 22/8 C: 21/9	E: 4.25 ± 1.02 m C: 3.92 ± 1.25 m	EA + mecobalamin tablets	30 min/session 6 sessions/week	4 weeks	Mecobalamin tablets	0.5 mg/time, tid	4 weeks	GSS NRS CMAP SNAP SNCV DML
Hadianfard et al., 2015	50/50	E: 25/25 C: 25/25	E: 44.5 ± 8.5 C: 42.5 ± 7.6	E: 24/1 C: 23/2	NI	MA + night splints	20 min/session 2 sessions/week	4 weeks	Ibuprofen + night splints	Ibuprofen: 400 mg/time, tid; Night splints: NI	Ibuprofen: 10 days; Night splints: 4 weeks	CTQ-SSS CTQ-FSS VAS MNCV DSL DML
Chung et al., 2016	181/181	E: 90/90 C: 91/91	E: 51 ± 10.2 C: 51 ± 8.7	E: 77/13 C: 81/10	E: 50 ± 52.7 m C: 51 ± 59.9 m	EA + night splints	20 min/session 1–2 sessions/week	17 weeks	Night splints	8 h/night, every night	17 weeks	CTQ-SSS CTQ-FSS DASH VAS
Maeda et al., 2017	51/43	E: 28/22 C: 23/21	E: 48.5 ± 10.1 C: 50.6 ± 7.8	E: 22/6 C: 20/3	E: 9.9 ± 8.9 y C: 9.4 ± 9.3 y	EA	20 min/session 1–3 weeks: 3 sessions/week 4–5 weeks: 2 sessions/week 6–8 weeks: 1 session/week	8 weeks	Sham acupuncture	20 min/session 1–3 weeks: 3 sessions/week 4–5 weeks: 2 sessions/week 6–8 weeks: 1 session/week	8 weeks	CTQ-SSS CTQ-FSS
Ural and Özturk, 2017	27/27	E: 14/14 C: 13/13	E: 50.5 ± 6.1 C: 51.5 ± 4.5	E: 14/0 C: 13/0	E: 18.3 ± 6.6 m C: 19.3 ± 11.1 m	MA + night splints	25 min/session 2–3 sessions/week	4 weeks	Night splints	NI	4 weeks	VAS DASH CMAP SNAP MNCV SNCV DML
Xie et al., 2018	86/86	E: 43/43 C: 43/43	E: 41.26 ± 6.78 C: 41.78 ± 6.49	E: 25/18 C: 24/19	E: 5.17 ± 3.48 m C: 4.89 ± 3.52 m	EA + medicine	40 min/session 5 sessions/week	4 weeks	Medicine (diclofenac sodium + mecobalamin tablets + vitamin B1 tablets + vitamin B6 tablets + bendazol tablets)	Diclofenac sodium: 25 mg/time, tid; Mecobalamin tablets: 0.5 mg/time, tid; Vitamin B1 tablets: 10 mg/time, tid; Vitamin B6 tablets: 10 mg/time, tid; Bendazol tablets: 10 mg/time, tid	Diclofenac sodium: 2 weeks; Mecobalamin/ vitamin B1/vitamin B6 tablets: 4 weeks	CTQ-SSS CTQ-FSS CMAP SNAP SNCV DML Responder rate
Tezel et al., 2019	51/44	E: 26/24 C: 25/20	E: 47.1 ± 7.7 C: 46.6 ± 8.1	E: 23/1 C: 19/1	NI	MA + night splints	20 min/session 2 sessions/week	5 weeks	Night splints	NI	5 weeks	CTQ-SSS CTQ-FSS VAS CAMP DML MNCV SNCV
Bahrami-Taghanaki et al., 2020	60/49	E: 30/25 C: 30/24	36.36 ± 7.74	NI	NI	MA + night splints	30 min/session 3 sessions/week	4 weeks	Celebrex tablets + night splints	Celebrex tablets: 100 mg/time, tid	4 weeks	GSS
Xiong, 2020	48/48	E: 24/24 C: 24/24	E: 46.3 ± 11.1 C: 49.2 ± 12.5	E: 16/8 C: 14/10	E: 2.7 ± 1.8 m C: 2.9 ± 1.5 m	EA + ultrashort wave therapy	30 min/session 6 sessions/week	6 weeks	Ultrashort wave therapy	20 min/session 6 sessions/week	6 weeks	CTQ-SSS Responder rate
Huang and Lin, 2022	50/50	E: 25/25 C: 25/25	E: 43.6 ± 6.5 C: 42.7 ± 7.6	E: 24/1 C: 23/2	NI	MA + night splints + ibuprofen	20 min/session 2 sessions/week	4 weeks	Night splints + ibuprofen	Ibuprofen: 400 mg, tid; Night splints: NI	Ibuprofen: 10 days; Night splints: 4 weeks	CTQ-SSS CTQ-FSS VAS DSL DML

E, experimental group; C, control group; min, minutes; m, months; h, hours; y, years; NI, no information; F, female; M, male; MA, manual acupuncture; EA, electroacupuncture; CTQ-SSS, Boston Carpal Tunnel Questionnaire-symptom severity scale; CTQ-FSS, CTQ-functional status scale; GSS, global symptoms score; DASH, disabilities of the arm, shoulder, and hand questionnaire; VAS, visual analog scale; NRS, numerical rating scales; CMAP, compound muscle action potential; SNAP, sensory nerve action potential; DML, distal motor latency; DSL, distal sensory latency; MNCV, motor nerve conduction velocity; SNCV, sensory nerve conduction velocity.

### 3.2. Risk of bias

**FIGURE 2:**
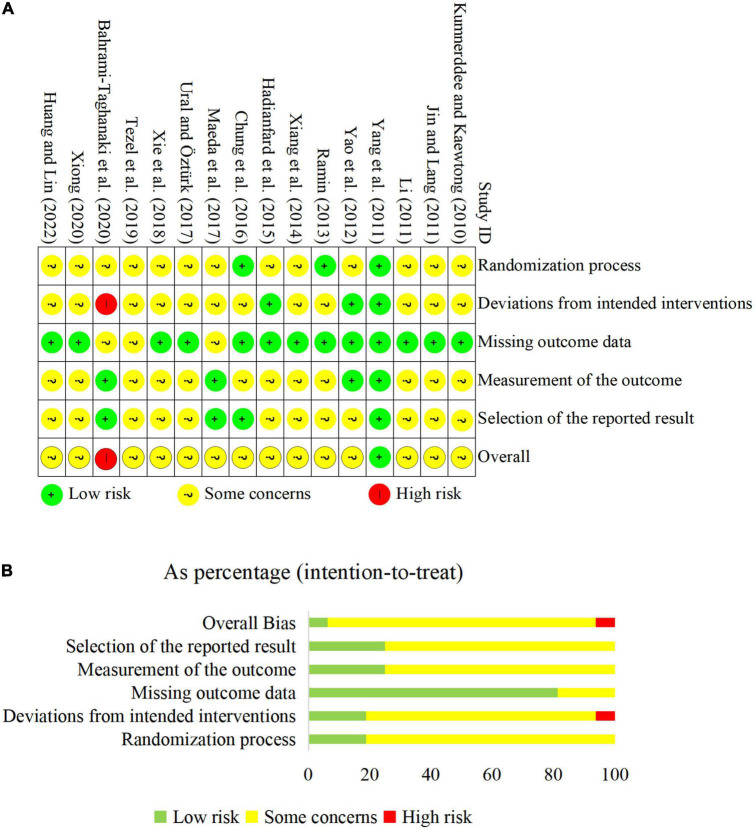
The results of risk of bias assessment. **(A)** Risk of bias of individual study; **(B)** overall risk of bias.

During the randomization process, 11 studies specified the randomization method (Kumnerddee and Kaewtong, 2010; Yang et al., 2011; Yao et al., 2012; Xiang et al., 2014; Hadianfard et al., 2015; Chung et al., 2016; Maeda et al., 2017; Ural and Özturk, 2017; Xie et al., 2018; Tezel et al., 2019; Bahrami-Taghanaki et al., 2020). Two studies (Yang et al., 2011; Chung et al., 2016) implemented appropriate methods to conceal the allocation sequence. All studies reported that there were comparable baselines between groups. Two studies (Yao et al., 2012; Maeda et al., 2017) blinded patients by conducting sham comparisons between the groups. Additionally, several outcomes, including symptom severity, functional status, and pain intensity, were participant-reported outcomes, which meant outcome assessors were blinded in the studies (Yao et al., 2012; Maeda et al., 2017). Seven studies (Kumnerddee and Kaewtong, 2010; Yang et al., 2011; Yao et al., 2012; Chung et al., 2016; Maeda et al., 2017; Tezel et al., 2019; Bahrami-Taghanaki et al., 2020) described dropouts rate with 1.6–18.3%; among these studies, four trials (Chung et al., 2016; Maeda et al., 2017; Tezel et al., 2019; Bahrami-Taghanaki et al., 2020) did not give the detailed reason of dropouts, and three studies (Yang et al., 2011; Yao et al., 2012; Chung et al., 2016) used intent-to-treat analysis. Four trials (Yang et al., 2011; Chung et al., 2016; Maeda et al., 2017; Bahrami-Taghanaki et al., 2020) provided the registration number or published protocol, and all of them reported planned outcomes. Overall, 1 RCT (Yang et al., 2011) was rated as having a low risk of bias, 14 (Kumnerddee and Kaewtong, 2010; Jin and Lang, 2011; Li, 2011; Yao et al., 2012; Ramin, 2013; Xiang et al., 2014; Hadianfard et al., 2015; Chung et al., 2016; Maeda et al., 2017; Ural and Özturk, 2017; Xie et al., 2018; Tezel et al., 2019; Xiong, 2020; Huang and Lin, 2022) had some concerns, and 1 (Bahrami-Taghanaki et al., 2020) was a high risk of bias. The results of the risk of bias in individual studies and the overall risk of bias are shown in Figure 2.

### 3.3. Acupuncture protocols included trials

**FIGURE 3:**
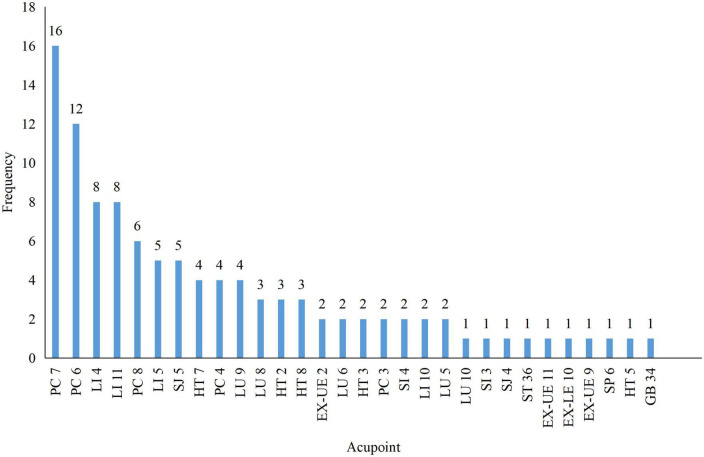
The frequency of acupoints selection.

There were different acupuncture techniques, among which manual acupuncture was applied in seven studies (Yang et al., 2011; Yao et al., 2012; Hadianfard et al., 2015; Ural and Özturk, 2017; Tezel et al., 2019; Bahrami-Taghanaki et al., 2020; Huang and Lin, 2022) and electroacupuncture in nine (Kumnerddee and Kaewtong, 2010; Jin and Lang, 2011; Li, 2011; Ramin, 2013; Xiang et al., 2014; Chung et al., 2016; Maeda et al., 2017; Xie et al., 2018; Xiong, 2020), respectively. All studies reported the selected acupoints, and the frequency of all acupoints is shown in Figure 3. The most used acupoints were Daling (PC7, 100%), Neiguan (PC6, 75.0%), Hegu (LI 4, 50.0%), Quchi (LI 11, 50.0%), and Laogong (PC 8, 37.5%). Thirteen included studies (Kumnerddee and Kaewtong, 2010; Jin and Lang, 2011; Yang et al., 2011; Yao et al., 2012; Ramin, 2013; Xiang et al., 2014; Hadianfard et al., 2015; Chung et al., 2016; Ural and Özturk, 2017; Tezel et al., 2019; Bahrami-Taghanaki et al., 2020; Xiong, 2020; Huang and Lin, 2022) applied the fixed acupoint protocol and 3 (Li, 2011; Maeda et al., 2017; Xie et al., 2018) used individualized acupoint protocol (fixed main acupoints plus acupoints based on syndrome differentiation). In addition, the retention time was mainly 20 or 30 min, and the total sessions ranged from 6 to 36 sessions within 20 days to 17 weeks of treatment duration.

### 3.4. STRICTA checklist for the included studies

According to the STRICTA checklist, the items with more than 70% of reporting rates were item 3a (number of treatment sessions, 100%), item 2e (needle stimulation, 100%), item 3b (frequency and duration of treatment sessions, 93.8%), item 6b (precise description of the control or comparator, 87.5%), item 2d (response sought, 81.3%), item 2f (needle retention time, 81.3%), and item 2g (needle type, 81.3%). Item 4b (setting and context of treatment) and 1c (the extent to which treatment was varied) were not reported in the included studies. Detailed information on the STRICTA checklist is provided in Supplementary material 3.

### 3.5. Primary outcomes

#### 3.5.1. Acupuncture as monotherapy

##### 3.5.1.1. Acupuncture vs. sham acupuncture

Maeda et al. (2017) found no difference in the improvement of symptom severity (CTQ-SSS) or functional status (CTQ-FSS) between the electroacupuncture and sham electroacupuncture groups.

##### 3.5.1.2. Acupuncture vs. night splints

Kumnerddee and Kaewtong (2010) found no difference in symptom severity (CTQ-SSS) or functional status (CTQ-FSS) between the electroacupuncture and night splints groups.

##### 3.5.1.3. Acupuncture vs. medicine

Yang et al. (2011) observed that manual acupuncture was not superior to prednisolone in reducing symptom severity as measured by CSS.

#### 3.5.2. Acupuncture as an adjunctive treatment

##### 3.5.2.1. Acupuncture plus night splints vs. sham acupuncture plus night splints

Yao et al. (2012) reported that there was no difference between the manual acupuncture plus night splints group and the sham acupuncture plus night splints group in symptom severity (CTQ-SSS) or functional status (CTQ-FSS).

##### 3.5.2.2. Acupuncture plus night splints vs. medicine plus night splints

**FIGURE 4:**
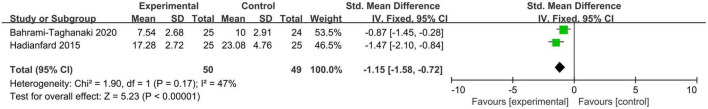
A meta-analysis of symptom severity of acupuncture plus night splints vs. medicine plus night splints.

Compared with medicine plus night splints, manual acupuncture plus night splints showed lower symptom severity (CTQ-SSS/GSS: SMD = −1.51, 95% CI −1.58 to −0.72, *I*^2^ = 47%) (Figure 4), but Hadianfard et al. (2015) found there was a greater effect of manual acupuncture plus night splints on functional status (CTQ-FSS).

##### 3.5.2.3. Acupuncture plus night splints vs. night splints

**FIGURE 5:**
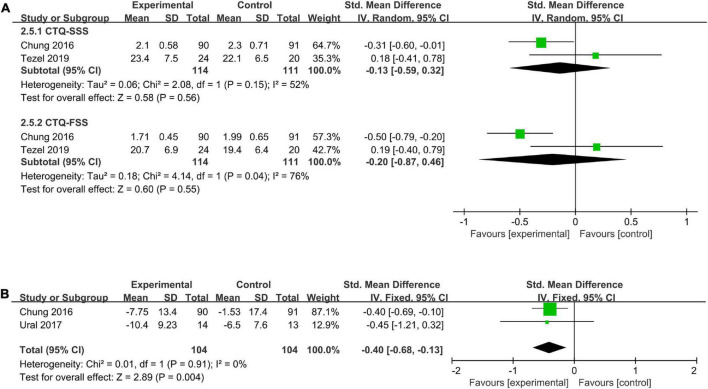
A meta-analysis of symptom severity and functional status of acupuncture plus night splints vs. night splints. **(A)** CTQ-SSS and CTQ-FSS; **(B)** change of DASH.

The results of the meta-analysis showed that neither symptom severity (CTQ-SSS: SMD = −0.13, 95% CI −0.59 to 0.32, *I*^2^ = 52%) nor functional status (CTQ-FSS: SMD = −0.20, 95% CI −0.87 to 0.46, *I*^2^ = 76%) was significantly different between the acupuncture plus the night splints group and the night splints group (Figure 5A). However, the improvement of functional status measured by DASH was greater in the acupuncture plus night splints group than in the night splints group (change of DASH: SMD = −0.40, 95% CI −0.68 to −0.13, *I*^2^ = 0%) (Figure 5B).

##### 3.5.2.4. Acupuncture plus medicine vs. medicine

**FIGURE 6:**
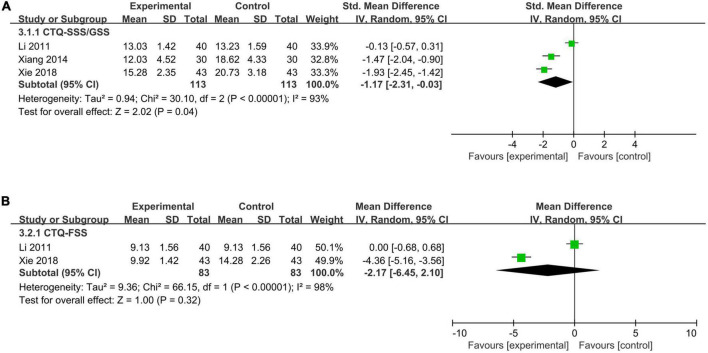
A meta-analysis of symptom severity and functional status of acupuncture plus medicine vs. medicine. **(A)** CTQ-SSS/GSS; **(B)** CTQ-FSS.

According to pooled results, the acupuncture plus medicine group had lower symptom severity (CTQ-SSS/GSS: SMD = −1.17, 95% CI −2.31 to −0.03, *I*^2^ = 93%) than the medicine group (Figure 6A), but the functional status (CTQ-FSS: MD = −2.17, 95% CI −6.45 to 2.10, *I*^2^ = 98%) was not significantly different between the two groups (Figure 6B).

##### 3.5.2.5. Acupuncture plus ultrashort wave therapy vs. ultrashort wave therapy

Xiong (2020) observed that patients who received acupuncture plus ultrashort wave therapy had lower symptom severity (CTQ-SSS) compared with those who received ultrashort wave therapy alone.

##### 3.5.2.6. Acupuncture plus medicine plus night splints vs. medicine plus night splints

One RCT (Huang and Lin, 2022) found that adjunctive manual acupuncture in addition to night splints and ibuprofen treatment could improve symptom severity (CTQ-SSS) and functional status (CTQ-FSS) better than night splints plus ibuprofen treatment.

### 3.6. Secondary outcomes (pain intensity)

#### 3.6.1. Acupuncture as monotherapy

Kumnerddee and Kaewtong (2010) reported that the electroacupuncture group showed a greater reduction in VAS than the night splints group.

#### 3.6.2. Acupuncture as adjuvant treatment

##### 3.6.2.1. Acupuncture plus night splints vs. medicine plus night splints

Hadianfard et al. (2015) observed that manual acupuncture plus night splints had a better effect than medicine plus night splints in decreasing VAS.

##### 3.6.2.2. Acupuncture plus night splints vs. night splints

**FIGURE 7:**
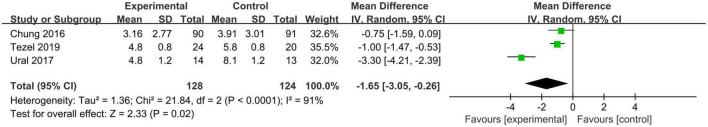
A meta-analysis of pain intensity of acupuncture plus night splints vs. night splints.

Meta-analysis results from three studies (Chung et al., 2016; Ural and Özturk, 2017; Tezel et al., 2019) showed that the acupuncture plus night splints group had lower pain intensity than the night splints group (VAS: MD = −1.65, 95% CI −3.05 to −0.26, *I*^2^ = 91%) (Figure 7).

##### 3.6.2.3. Acupuncture plus medicine vs. medicine

Xiang et al. (2014) suggested that electroacupuncture plus medicine treatment was superior to medical treatment in relieving pain as measured by the NRS.

##### 3.6.2.4. Acupuncture plus medicine plus night splints vs. medicine plus night splints

Huang and Lin (2022) found that manual acupuncture plus medicine and night splints were more effective in improving VAS scores than medicine plus night splints.

### 3.7. Secondary outcomes (electrophysiological parameters)

The results for electrophysiological parameters are shown in Table 2.

**TABLE 2:** The results of electrophysiological parameters for all comparisons.

Comparison	Experimental group vs. control group	Outcomes	Number of studies	Intergroup differences		Heterogeneity (*I*^2^)
				**MD (95% CI)**	***P***	
Acupuncture as monotherapy	Acupuncture vs. medicine	CMAP (mv)	3	−1.02 (−2.02 to −0.03)	0.04	46%
		DML (ms)	3	−0.31 (−0.96 to 0.34)	0.35	75%
		MNCV (m/s)	2	−3.57 (−13.79 to 6.65)	0.49	92%
		SNAP (μv)	2	−3.14 (−6.84 to 0.56)	0.10	0%
		SNCV (m/s)	3	−1.12 (−6.39 to 4.14)	0.68	79%
		DSL (ms)	2	−0.05 (−0.78 to 0.69)	0.90	84%
Acupuncture as adjunctive therapy	Acupuncture plus medicine vs. medicine	CMAP (mv)	3	2.30 (0.84 to 3.77)	0.002	81%
		DML (ms)	3	−0.47 (−0.66 to −0.28)	<0.00001	32%
		SNCV (m/s)	3	4.02 (2.44 to 5.59)	<0.00001	0%
		SNAP (μv)	3	2.53 (1.63 to 3.44)	<0.00001	0%
	Acupuncture plus night splints vs. night splints	CMAP (mv)	2	1.31 (−1.04 to 3.66)	0.27	58%
		DML (ms)	2	0.05 (−0.33 to 0.43)	0.79	0%
		MNCV (m/s)	2	1.81 (−0.55 to 4.18)	0.13	0%
		SNCV (m/s)	2	0.24 (−2.20 to 2.67)	0.85	0%
		SNAP (μv)	1	3.20 (−0.73 to 7.13)	0.11	–
	Acupuncture plus medicine plus night splints vs. medicine plus night splints	DML (ms)	1	−0.22 (−0.48 to 0.04)	0.09	–
		DSL (ms)	1	−0.53 (−0.75 to −0.31)	<0.00001	–
	Acupuncture plus night splints vs. Medicine plus night splints	DML (ms)	1	−0.20 (−0.43 to 0.03)	0.09	–
		MNCV (m/s)	1	1.76 (0.68 to 2.84)	0.001	–
		DSL (ms)	1	−0.26 (−0.37 to −0.15)	<0.00001	–

CMAP, compound muscle action potential; SNAP, sensory nerve action potential; DML, distal motor latency; DSL, distal sensory latency; MNCV, motor nerve conduction velocity; SNCV, sensory nerve conduction velocity.

#### 3.7.1. Acupuncture as monotherapy

Compared with medicine, the acupuncture group had a lower CMAP (MD = −1.02, 95% CI −2.02 to −0.03, *I*^2^ = 46%). No differences were found in DML, DSL, MNCV, SNAP, and SNCV between the two groups.

#### 3.7.2. Acupuncture as an adjunctive treatment

##### 3.7.2.1. Acupuncture plus night splints vs. medicine plus night splints

Hadianfard et al. (2015) found a faster MNCV and shorter DSL in the acupuncture plus night splints group than in the medicine plus night splints group. However, no difference in DML existed between the two groups.

##### 3.7.2.2. Acupuncture plus night splints vs. night splints

There were no differences between the acupuncture plus night splints group and the night splints group in CMAP, DML, MNCV, SNCV, and SNAP.

##### 3.7.2.3. Acupuncture plus medicine vs. medicine

Compared with the medicine group, the acupuncture plus medicine group showed higher CMAP (MD = 2.30, 95% CI 0.84 to 3.77, *I*^2^ = 81%) and SNAP (MD = 2.53, 95% CI 1.63 to 3.44, *I*^2^ = 0%), shorter DML (MD = −0.47, 95% CI −0.66 to −0.28, *I*^2^ = 32%), and faster SNCV (MD = 4.02, 95% CI 2.44 to 5.59, *I*^2^ = 0%).

##### 3.7.2.4. Acupuncture plus medicine and night splints vs. medicine plus night splints

Huang and Lin (2022) found that the DML showed no significant difference between the acupuncture plus medicine and night splints group and the medicine plus night splints group, but the DSL was shorter in the acupuncture plus medicine and night splints group.

### 3.8. Responder rate

Three studies (Jin and Lang, 2011; Xie et al., 2018; Xiong, 2020) provided the responder rate. Jin and Lang (2011) reported a comparable responder rate between the acupuncture group and the medicine group. Xie et al. (2018) reported a superior responder rate in the acupuncture plus medicine group than the medicine group. Xiong (2020) observed that acupuncture plus ultrashort wave therapy had no better than ultrashort wave therapy in responder rate.

### 3.9. Adverse events

Four studies (Jin and Lang, 2011; Yang et al., 2011; Yao et al., 2012; Tezel et al., 2019) found no adverse events or serious adverse events related to acupuncture treatment occured. Kumnerddee and Kaewtong (2010) observed 6 of 30 cases in electroacupuncture group experienced skin bruises but no serious complication took place. Another study (Chung et al., 2016) reported electroacupuncture-related adverse events, including bruises at acupoints (4/90), mild local dermatitis around acupoints (3/90), increased pain (2/90), and numbness and tingling after electroacupuncture treatment (2/90), and the above adverse events disappeared within a week. The rest of the 10 studies provided no information on the adverse events.

### 3.10. Certainty of evidence

There was low and very low certainty of evidence attributed to some concern risk of bias, imprecision, and strongly suspected publication bias. A summary of the finding table is provided in Supplementary material 4.

## 4. Discussion

### 4.1. Summary of main results

We included 16 RCTs with 1,025 subjects and explored the effect of acupuncture as monotherapy and adjunctive therapy on CTS. Compared with night splints, acupuncture alone was more effective in relieving pain, but there were no differences in symptom severity and functional status. Acupuncture had no advantage over medicine in improving symptom severity or electrophysiological parameters. As an adjunctive treatment, acupuncture might effectively alleviate symptom severity, functional status, pain intensity, and electrophysiological parameters.

Meanwhile, acupuncture as adjunctive therapy was more effective than medicine to ameliorate symptom severity, functional status, pain intensity, and electrophysiological parameters. According to narrative analysis, acupuncture as monotherapy or adjunctive therapy, showed no superiority to sham acupuncture. Few acupuncture-related adverse events were reported. The above evidence had low or very low certainty.

### 4.2. Compared with previous reviews

Sim et al. (2011) included six RCTs and published the first systematic review of acupuncture for CTS, but three of the RCTs they identified were excluded from our study because the participants received other traditional Chinese medicine in one RCT (Shi et al., 2006) and cointerventions between groups were not comparable in the other two RCTs (Hu et al., 2000; Cai, 2007). Limited by insufficient RCTs, Sim et al. (2011) summarized the evidence of acupuncture for CTS as encouraging but not convincing. Choi et al. (2018) also found insufficient evidence to assess the effect of acupuncture and related interventions on CTS with 12 identified RCTs. Wu et al. (2020) conducted the latest systematic review involving 10 RCTs. Except for manual and electroacupuncture, they included laser acupuncture, moxibustion, and transcutaneous electrical nerve stimulation treatment. They drew the conclusion that acupuncture and related therapies appeared to be effective in improving symptoms, function, and pain in CTS, and emphasized that the validity of such a conclusion was limited. We included 16 RCTs to update the evidence and investigate the effect of acupuncture as monotherapy or adjunctive therapy for CTS.

### 4.3. The effect of acupuncture on CTS

The narrative analysis showed that neither acupuncture alone nor acupuncture as a adjunctive treatment had superiority over sham acupuncture. However, these results were derived from two independent studies, respectively (Yao et al., 2012; Maeda et al., 2017). Given the limited studies and risk of the underrated effect of acupuncture in sham-control trials with a small sample size (Lundeberg et al., 2008; Birch et al., 2022a,b), we failed to identify the advantage of acupuncture over sham acupuncture for CTS, which should continue to be explored in future studies.

One included RCT showed that 10-session acupuncture alone might be more effective than night splints in relieving pain intensity but not symptom severity or functional status. Night splints are recommended for CTS to improve short-term symptoms and function (Erickson et al., 2019). Whether there is a different long-term effect between acupuncture and night splints is unknown. No clear advantages of acupuncture as a monotherapy were observed compared with medicine. Among comparative medicines used in included studies, such as prednisolone (Yang et al., 2011; Ramin, 2013) and oral vitamin B12 (Jin and Lang, 2011), only prednisolone was recommended by the AAOS. Yang et al. (2011) found that patients with CTS who received acupuncture had a lower recurrence rate than those who received prednisolone in the 1-year follow-up period, which indicated acupuncture might have a better long-term effect than prednisolone. Due to insufficient studies, we were unable to compare the effect of acupuncture with other active treatments. More relevant head-to-head trials should be conducted in the future to focus on clinical and cost effects.

Patients with CTS who received acupuncture plus other treatment(s) showed more improvement in symptoms, function, or pain. However, these positive findings of acupuncture as adjunctive therapy came from open-label RCTs, which could be influenced by the patients’ subjective intentions. Based on the results of electrophysiological parameters, we found acupuncture combined with medicine could improve median nerve function better than medicine alone, which provided objective evidence for the adjunctive effect of acupuncture. However, the adjunctive effect of acupuncture should be further investigated in clinical trials with objective outcomes.

### 4.4. Implications for future research

The outcomes that were measured by subjective tools, such as CTQ, GSS, DASH, and VAS, relied on participants’ self-reports, which might induce measurement bias favoring acupuncture in open-label studies. Therefore, studies using objective outcomes are vital to build convincing evidence of acupuncture for CTS. According to the ROB 2 assessment, allocation concealment and advanced registration, or protocol, should be improved to enhance the credibility of the evidence. Meanwhile, in compliance with the STRICTA, authors should take care to report the details of the intervention, especially in items of acupuncture rationale, cointerventions, practitioner background and control or comparator interventions.

### 4.5. Limitations

Our systematic review and meta-analysis included the latest RCTs and assessed the effect of acupuncture on CTS. However, several limitations exist and should be considered. In the present review, the small sample size, substantial heterogeneity, and potential risk of bias of the included studies reduced the certainty of the evidence. Thus, the findings should be treated with caution. Owing to limited RCTs and data, we failed to investigate the advantages of different acupuncture techniques, identify the optimal parameters of the acupuncture protocol, or explore the follow-up effect of acupuncture.

## 5. Conclusion

Acupuncture as an adjunctive treatment may be effective for patients with CTS. In addition, more rigorous studies with objective outcomes are needed to investigate the effect of acupuncture in contrast with sham acupuncture or other active treatments.

## Data availability statement

The original contributions presented in this study are included in this article/Supplementary material, further inquiries can be directed to the corresponding authors.

## Author contributions

RJ and HF conceptualized the study and provided methodological support. PY and GC selected the studies. JL and JD extracted the data. QD and XL assessed the risk of bias and wrote and edited the manuscript. HF and QD assessed the reporting quality of the intervention. FW and YY evaluated the grade. All authors contributed to the article and approved the submitted version.

## References

[B1] Atroshi I. Gummesson C. Johnsson R. Ornstein E. Ranstam J. Rosén I. (1999). Prevalence of carpal tunnel syndrome in a general population. *JAMA* 282 153–158. 10.1001/jama.282.2.153 10411196

[B2] Bahrami-Taghanaki H. Azizi H. Hasanabadi H. Jokar M. H. Iranmanesh A. Khorsand-Vakilzadeh A. (2020). Acupuncture for carpal tunnel syndrome: A randomized controlled trial studying changes in clinical symptoms and electrodiagnostic tests. *Altern. Ther. Health Med.* 26 10–16. 31634868

[B3] Birch S. Lee M. S. Kim T. H. Alraek T. (2022a). Historical perspectives on using sham acupuncture in acupuncture clinical trials. *Integr. Med. Res.* 11:100725. 10.1016/j.imr.2021.100725 34458094 PMC8379290

[B4] Birch S. Lee M. S. Kim T. H. Alraek T. (2022b). On defining acupuncture and its techniques: A commentary on the problem of sham. *Integr. Med. Res.* 11:100834. 10.1016/j.imr.2022.100834 35111572 PMC8790499

[B5] Cai D. F. (2007). Effect of warming acupuncture and manual release on carpal tunnel syndrome. *Inf. Trad. Chin. Med.* 5 56–57.

[B6] Calandruccio J. H. Thompson N. B. (2018). Carpal tunnel syndrome: Making evidence-based treatment decisions. *Orthop. Clin. North Am.* 49 223–229. 10.1016/j.ocl.2017.11.009 29499823

[B7] Chesterton L. S. Blagojevic-Bucknall M. Burton C. Dziedzic K. S. Davenport G. Jowett S. M. (2018). The clinical and cost-effectiveness of corticosteroid injection versus night splints for carpal tunnel syndrome (INSTINCTS trial): An open-label, parallel group, randomised controlled trial. *Lancet* 392 1423–1433. 30343858 10.1016/S0140-6736(18)31572-1 PMC6196880

[B8] Choi G. H. Wieland L. S. Lee H. Sim H. Lee M. S. Shin B. C. (2018). Acupuncture and related interventions for the treatment of symptoms associated with carpal tunnel syndrome. *Cochrane Database Syst. Rev.* 12:Cd011215. 10.1002/14651858.CD011215.pub2 30521680 PMC6361189

[B9] Chung V. C. H. Ho R. S. T. Liu S. Chong M. K. C. Leung A. W. N. Yip B. H. K. (2016). Electroacupuncture and splinting versus splinting alone to treat carpal tunnel syndrome: A randomized controlled trial. *CMAJ Can. Med. Assoc. J.* 188 867–875. 10.1503/cmaj.151003 27270119 PMC5008933

[B10] Daniell W. E. Fulton-Kehoe D. Franklin G. M. (2009). Work-related carpal tunnel syndrome in Washington State workers’ compensation: Utilization of surgery and the duration of lost work. *Am. J. Ind. Med.* 52 931–942. 10.1002/ajim.20765 19882743

[B11] Erickson M. Lawrence M. Jansen C. W. S. Coker D. Amadio P. Cleary C. (2019). Hand pain and sensory deficits: Carpal tunnel syndrome. *J. Orthop. Sports Phys. Ther.* 49 CG1–CG85. 10.2519/jospt.2019.0301 31039690

[B12] Feng B. Chen K. Zhu X. Ip W. Y. Andersen L. L. Page P. (2021). Prevalence and risk factors of self-reported wrist and hand symptoms and clinically confirmed carpal tunnel syndrome among office workers in China: A cross-sectional study. *BMC Public Health* 21:57. 10.1186/s12889-020-10137-1 33407293 PMC7789363

[B13] Graham B. Peljovich A. E. Afra R. Cho M. S. Gray R. Stephenson J. (2016). The American academy of orthopaedic surgeons evidence-based clinical practice guideline on: Management of carpal tunnel syndrome. *J. Bone Joint Surg. Am.* 98 1750–1754. 10.2106/JBJS.16.00719 27869627

[B14] Hadianfard M. Bazrafshan E. Momeninejad H. Jahani N. (2015). Efficacies of acupuncture and anti-inflammatory treatment for carpal tunnel syndrome. *J. Acupunct. Meridian Stud.* 8 229–235. 10.1016/j.jams.2014.11.005 26433799

[B15] Hu N. W. Liu J. Y. Wang F. M. (2000). Clinical observation of combination of acupuncture and medicine in treating carpal tunnel syndrome. *Acta Chin. Med. Pharmacol.* 3 57–58.

[B16] Huang X. X. Lin C. J. (2022). Preliminary analysis of the effect of conventional treatment combined with acupuncture on mild to moderate carpal tunnel syndrome. *Fujian Med. J.* 44 63–66.

[B17] Jin L. Q. Lang B. X. (2011). Effect of electroacupuncture plus acupoint injection in treating carpal tunnel syndrome of early stage. *Shanghai J. Acupunct. Moxibust.* 30 464–466.

[B18] Kumnerddee W. Kaewtong A. (2010). Efficacy of acupuncture versus night splinting for carpal tunnel syndrome: A randomized clinical trial. *J. Med. Assoc. Thai.* 93 1463–1469. 21344811

[B19] Li M. (2011). *Study on the Electrophysiological Assessment of the Efficacyof Electric Acupuncture in Treatment of Mild and Moderate Carpal Tunnel Syndromes.* Master’s thesis. Guangzhou: Guangzhou University of Chinese Medicine.

[B20] Lundeberg T. Lund I. Näslund J. Thomas M. (2008). The emperors sham – wrong assumption that sham needling is sham. *Acupunct. Med.* 26 239–242. 10.1136/aim.26.4.239 19098696

[B21] MacPherson H. Altman D. G. Hammerschlag R. Youping L. Taixiang W. White A. (2010). Revised STandards for Reporting Interventions in Clinical Trials of Acupuncture (STRICTA): Extending the CONSORT statement. *J. Altern. Complement. Med.* 16 St1–St14. 10.1089/acm.2010.1610 20954957

[B22] Maeda Y. Kim H. Kettner N. Kim J. Cina S. Malatesta C. (2017). Rewiring the primary somatosensory cortex in carpal tunnel syndrome with acupuncture. *Brain* 140 914–927. 10.1093/brain/awx015 28334999 PMC5837382

[B23] Manente G. Torrieri F. Di Blasio F. Staniscia T. Romano F. Uncini A. (2001). An innovative hand brace for carpal tunnel syndrome: A randomized controlled trial. *Muscle Nerve* 24 1020–1025. 10.1002/mus.1105 11439376

[B24] Padua L. Coraci D. Erra C. Pazzaglia C. Paolasso I. Loreti C. (2016). Carpal tunnel syndrome: Clinical features, diagnosis, and management. *Lancet Neurol.* 15 1273–1284. 10.1016/S1474-4422(16)30231-9 27751557

[B25] Page M. J. Massy-Westropp N. O’Connor D. Pitt V. (2012). Splinting for carpal tunnel syndrome. *Cochrane Database Syst. Rev.* 2012:Cd010003. 10.1002/14651858.CD010003 22786532 PMC7389822

[B26] Page M. J. McKenzie J. E. Bossuyt P. M. Boutron I. Hoffmann T. C. Mulrow C. D. (2021). The PRISMA 2020 statement: An updated guideline for reporting systematic reviews. *BMJ* 372 n71. 10.1136/bmj.n71 33782057 PMC8005924

[B27] Palmer D. H. Hanrahan L. P. (1995). Social and economic costs of carpal tunnel surgery. *Instr. Course Lect.* 44 167–172. 7797856

[B28] Qiao L. Guo M. Qian J. Xu B. Gu C. Yang Y. (2020). Research advances on acupuncture analgesia. *Am. J. Chin. Med.* 48 245–258. 10.1142/S0192415X20500135 32138535

[B29] Ramin M. (2013). *Comparison of Acupuncture and Corticosteroid in lmprovement of Carpal Tunnel Syndrome and its mechanism.* Ph.D. thesis. Nanjing: Nanjing University of Chinese medicine.

[B30] Shea B. J. Reeves B. C. Wells G. Thuku M. Hamel C. Moran J. (2017). AMSTAR 2: a critical appraisal tool for systematic reviews that include randomised or non-randomised studies of healthcare interventions, or both. *BMJ* 358 j4008. 10.1136/bmj.j4008 28935701 PMC5833365

[B31] Shi Q. MacDermid J. C. (2011). Is surgical intervention more effective than non-surgical treatment for carpal tunnel syndrome? A systematic review. *J. Orthop. Surg. Res.* 6:17. 10.1186/1749-799X-6-17 21477381 PMC3080334

[B32] Shi Y. S. Fang W. Zhao X. Y. Li H. X. Liu S. (2006). Control study on effect of pricking collateral blood therapy combined with massage on mild carpal tunnel syndrome. *Chin. J. Integr. Trad. West. Med.* 6 497–499. 16841662

[B33] Sim H. Shin B. C. Lee M. S. Jung A. Lee H. Ernst E. (2011). Acupuncture for carpal tunnel syndrome: a systematic review of randomized controlled trials. *J. Pain.* 12 307–314. 10.1016/j.jpain.2010.08.006 21093382

[B34] Spahn G. Wollny J. Hartmann B. Schiele R. Hofmann G. O. (2012b). Meta-analysis for the evaluation of risk factors for carpal tunnel syndrome (CTS) Part II. Occupational risk factors. *Z. Orthop. Unfall* 150 516–524. 10.1055/s-0032-1315346 23076750

[B35] Spahn G. Wollny J. Hartmann B. Schiele R. Hofmann G. O. (2012a). Meta-analysis for the evaluation of risk factors for carpal tunnel syndrome (CTS) Part I. General factors. *Z. Orthop. Unfall* 150 503–515. 10.1055/s-0032-1315345 23076749

[B36] Tezel N. Umay E. Yilmaz V. Cakci A. (2019). Acupuncture plus night splint for quality of life and disability in patients with carpal tunnel syndrome: a randomized controlled trial. *Integr. Med. Res.* 8 284–288. 10.1016/j.imr.2019.11.003 31828011 PMC6889040

[B37] Ural F. G. Özturk G. T. (2017). The acupuncture effect on median nerve morphology in patients with carpal tunnel syndrome: an ultrasonographic study. *Evid. Based Complement. Altern. Med.* 2017:7420648. 10.1155/2017/7420648 28676832 PMC5476875

[B38] Wipperman J. Goerl K. (2016). Carpal tunnel syndrome: diagnosis and management. *Am. Fam. Physic.* 94 993–999. 28075090

[B39] Wu I. X. Lam V. C. Ho R. S. Cheung W. K. Sit R. W. Chou L. W. (2020). Acupuncture and related interventions for carpal tunnel syndrome: systematic review. *Clin. Rehabil.* 34 34–44. 10.1177/0269215519877511 31556315

[B40] Xiang Y. Jiang H. Li J. H. (2014). Effect of electroacupuncture on mild to moderate carpal tunnel syndrome. *Clin. Educ. Gen. Pract.* 12 684–686.

[B41] Xie Q. E. Pan J. Zhang X. Xu Y. G. Wang L. H. (2018). Effect of electroacupuncture on patients with mild to moderate carpal tunnel syndrome and electrophysiological parameters. *Prog. Mod. Biomed.* 18 343–347.

[B42] Xiong P. (2020). Effect of electroacupuncture combined with local acupoint selection on carpal tunnel syndrome. *Hubei J. Trad. Chin. Med.* 42 52–54. 30521680

[B43] Yang C. P. Wang N. H. Li T. C. Hsieh C. L. Chang H. H. Hwang K. L. (2011). A randomized clinical trial of acupuncture versus oral steroids for carpal tunnel syndrome: a long-term follow-up. *J. Pain* 12 272–279. 10.1016/j.jpain.2010.09.001 21111685

[B44] Yao E. Gerritz P. K. Henricson E. Abresch T. Kim J. Han J. (2012). Randomized controlled trial comparing acupuncture with placebo acupuncture for the treatment of carpal tunnel syndrome. *PM R* 4 367–373. 10.1016/j.pmrj.2012.01.008 22405683

