# Supplementary material for: Acupuncture for carpal tunnel syndrome: A systematic review and meta-analysis of randomized controlled trials
Source: Front Neurosci. 2023 Feb 23;17:1097455. doi: 10.3389/fnins.2023.1097455 (PMC9995832; doi:10.3389/fnins.2023.1097455)
Supplement: Supplementary file 1 [file Table_1.DOC]

**Search strategies of all databases**

| **Database** | **Search strategies** |
| --- | --- |
| PubMed | (Acupuncture[Mesh] OR Acupuncture Therapy[Mesh] OR Acupuncture Analgesia[MESH] OR acupuncture [TIAB] OR electroacupuncture [TIAB] OR electro-acupuncture[TIAB] OR pharmacopuncture [TIAB] OR (chinese medicine[TIAB] AND needl*[TIAB]) OR acupoint*[TIAB] OR acupressure[TIAB]) AND  (Carpal Tunnel Syndrome[Mesh] OR (carpal[TIAB] AND tunnel[TIAB] AND syndrome*[TIAB]) OR carpal tunnel[TIAB] OR ((median neuropath*[TIAB] OR nerve entrapment[TIAB] OR nerve compression[TIAB] OR compression neuropath*[TIAB] OR entrapment neuropath*[TIAB]) AND carpal[TIAB])) |
| EMbase | #1 'carpal tunnel syndrome'/exp OR 'carpal tunnel syndrome':ab,ti OR 'carpal tunnel':ab,ti OR (carpal:ab,ti AND tunnel:ab,ti AND syndrome*:ab,ti) OR (('median neuropath*':ab,ti OR 'nerve entrapment':ab,ti OR 'nerve compression':ab,ti OR 'compression neuropath*':ab,ti OR 'entrapment neuropath*':ab,ti) AND carpal:ab,ti)  #2 'acupuncture'/exp OR 'acupuncture analgesia'/exp OR acupuncture:ab,ti OR electroacupuncture:ab,ti OR 'electro acupuncture':ab,ti OR pharmacopuncture:ab,ti OR acupoint*:ab,ti OR acupressure:ab,ti OR ('chinese medicine':ab,ti AND needl*:ab,ti)  #3 #1 AND #2 |
| The Cochrane library | #1 MeSH descriptor: [Acupuncture Analgesia] explode all trees  #2 MeSH descriptor: [Acupuncture] explode all trees  #3 MeSH descriptor: [Acupuncture Therapy] explode all trees  #4 (acupuncture):ti,ab,kw OR (electroacupuncture):ti,ab,kw OR (electro-acupuncture):ti,ab,kw OR (pharmacopuncture):ti,ab,kw OR (acupressure):ti,ab,kw  #5 (acupoint*):ti,ab,kw  #6 (chinese medicine):ti,ab,kw AND (needl*):ti,ab,kw  #7 #1 OR #2 OR #3 OR #4 OR #5 OR #6  #8 (carpal):ti,ab,kw AND (tunnel):ti,ab,kw AND (syndrome*):ti,ab,kw  #9 MeSH descriptor: [Carpal Tunnel Syndrome] explode all trees  #10 (carpal tunnel):ti,ab,kw  #11 ((median neuropath*):ti,ab,kw OR (nerve entrapment):ti,ab,kw OR (nerve compression):ti,ab,kw OR (compression neuropath*):ti,ab,kw OR (entrapment neuropath*):ti,ab,kw) AND (carpal):ti,ab,kw  #12 #8 OR #9 OR #10 OR #11  #13 #7 AND #12 |
| China National Knowledge Infrastructure (CNKI) | TKA = (针灸 + 针刺 + 针灸疗法 + 针刺疗法 + 电针 + 手针 + 穴位 + 腧穴) AND TKA = (腕管综合征 + 鼠标手 + 腕管狭窄征 + 腕管狭窄 + 腕管) |
| Wanfang Database | #1 主题:(针灸) or 主题:(针刺) or 主题:(针灸疗法) or 主题:(针刺疗法) or 主题:(电针) or 主题:(手针) or 主题:(穴位) or 主题:(腧穴)  #2 主题:(腕管综合征) or 主题:(鼠标手) or 主题:(腕管狭窄征) or 主题:(腕管狭窄) or 主题:(腕管)  #3 #1 AND #2 |
| Chinese Science and Technology Periodical Database (VIP) | (M=腕管综合征 OR M=鼠标手 OR M=腕管狭窄征 OR M= 腕管狭窄 OR M=腕管) AND (M=针灸 OR M=针刺 OR M=针灸疗法 OR M=针刺疗法 OR M=电针 OR M=手针 OR M=穴位 OR M=腧穴) |
| Chinese Biomedical Literature Database (CBM) | #1 "针灸疗法"[不加权:扩展]  #2 "针灸"[常用字段:智能] OR "针刺"[常用字段:智能] OR "针灸疗法"[常用字段:智能] OR "针刺疗法"[常用字段:智能] OR "手针"[常用字段:智能] OR "电针"[常用字段:智能] OR "腧穴"[常用字段:智能] OR "穴位"[常用字段:智能]  #3 "腕管综合征"[不加权:扩展]  #4 "腕管综合征"[常用字段:智能] OR "腕管"[常用字段:智能] OR "鼠标手"[常用字段:智能] OR "腕管狭窄征"[常用字段:智能] OR "腕管狭窄"[常用字段:智能]  #5 #2 OR #1  #6 #4 OR #3 |
