# Supplementary material for: Acupuncture for carpal tunnel syndrome: A systematic review and meta-analysis of randomized controlled trials
Source: Front Neurosci. 2023 Feb 23;17:1097455. doi: 10.3389/fnins.2023.1097455 (PMC9995832; doi:10.3389/fnins.2023.1097455)
Supplement: Supplementary file 2 [file Table_2.DOCX]

**List of excluded studies**

**Non-randomized controlled trials (n= 21)**

1. Zhao ZW. Acupuncture for Carpal Tunnel Syndrome. *Shanghai Journal of Acupuncture and Moxibustion* (1991) 10(2). Epub 19941231.

2. Wang AJ. Acupuncture Treatment of Carpal Tunnel Syndrome in 57 Cases. *Shanghai Journal of Acupuncture and Moxibustion* (1992) (01):46. doi: 10.13460/j.issn.1005-0957.1992.01.035.

3. Chen N. Acupuncture Treatment of Carpal Tunnel Syndrome in 98 Cases. *Jiangsu Journal of Traditional Chinese Medicine* (1995) 16(2). Epub 19951231.

4. Qi LZ. Treatment of Carpal Tunnel Syndrome by Embedding Acupuncture at Daling Point. *Shanghai Journal of Acupuncture and Moxibustion* (1995) 14(1). Epub 19951231.

5. Cai LQ. Clinical Report of 54 Cases of Carpal Tunnel Syndrome Treated by "Guan" Acupuncture. *Journal of Clinical Acupuncture and Moxibustion* (1996) 12(12). Epub 19961231.

6. Wang DY, Zhang B, Gao FW. Electroacupuncture for Carpal Tunnel Syndrome in 25 Cases. *Chinese Acupuncture & Moxibustion* (1996) 16(5). Epub 19961231.

7. Chen X. Acupotomy for Carpal Tunnel Syndrome. *Fujian Journal of Traditional Chinese Medicine* (1998) 29(3). Epub 19981231.

8. Xu CG. Acupotomy for Carpal Tunnel Syndrome in 26 Cases. *Shandong Journal of Traditional Chinese Medicine* (1999) 18(12). Epub 19991231.

9. Xu WL, Xu X. Warm Acupuncture for Carpal Tunnel Syndrome. *Clinical Journal of Traditional Chinese Medicine* (1999) 11(3). Epub 19991231.

10. Hu LF. Treatment of Carpal Tunnel Syndrome by Acupuncture and Massage in 46 Cases. *China's Naturopathy* (2002) 10(9). Epub 20021231.

11. Peng JH. 48 Cases of Carpal Tunnel Syndrome Treated by Flat Acupuncture and Unidirectional Twist Acupuncture. *Chinese Acupuncture & Moxibustion* (2002) 22(8). Epub 20021231.

12. Chen Y. Acupuncture Combined with Tdp Irradiation for Carpal Tunnel Syndrome in 96 Cases. *Journal of Practical Traditional Chinese Medicine* (2006) 22(3). Epub 20060430.

13. Schulman RA, Liem B, Moroz A. Treatment of Carpal Tunnel Syndrome with Medical Acupuncture (Medical Acupuncture 20, 3, (163-167)). *Medical Acupuncture* (2008) 20(4):301. doi: 10.1089/acu.2008.703.cxn.

14. Sun PY, Guo FQ, Zhang FP, Ma RZ. 30 Cases of Carpal Tunnel Syndrome Treated by Acupuncture and Moxibustion with Wormwood Box. *Clinical Journal of Acupuncture* (2008) 24(4). Epub 20080720.

15. Xiang AL, Cao QL, Deng XH. Treatment of Carpal Tunnel Syndrome by Laser Acupotomy and Nursing Care. *Journal of Yunyang Medical College* (2008) 27(5). Epub 20090130.

16. Chu XB, Tian K, Tong PJ. Mikebao Acupoint Injection Improves Nerve Dysfunction in Patients with Advanced Carpal Tunnel Syndrome. *The Journal of Traditional Chinese Orthopedics and Traumatology* (2009) 21(3). Epub 20090615.

17. Pan JA. 86 Cases of Carpal Tunnel Syndrome Were Treated by Shixuan Point. *Shanghai Journal of Acupuncture and Moxibustion* (2010) 29(3). Epub 20100530.

18. Li GD. 40 Cases of Carpal Tunnel Syndrome Treated by Integrated Chinese and Western Medicine. *Practical Clinical Journal of Integrated Traditional Chinese and Western Medicine* (2011) 11(2). Epub 20110820.

19. Zhang L. Acupuncture and Infrared Irradiation for Carpal Tunnel Syndrome. *Guangming Journal of Chinese Medicine* (2011) 26(7). Epub 20111130.

20. Zeng Y. Treatment of 58 Cases of Carpal Tunnel Syndrome with Needling and Electroacupuncture. *Journal of Medical Information* (2014) (27). Epub 20200315.

21. Pei LQ, Wen H. 31 Cases of Mild to Moderate Carpal Tunnel Syndrome Were Treated by Combined Acupuncture of Du Vein and Back Shu Point. *Chinese Acupuncture & Moxibustion* (2022):1-3. doi: 10.13703/j.0255-2930.20220429-0003.

**Ineligible interventions (n= 14)**

1. Hu NW, Liu JY, Wang FM. Clinical Observation of Combination of Acupuncture and Medicine in Treating Carpal Tunnel Syndrome. *Acta Chinese Medicine and Pharmacology* (2000) 28(3). Epub 20001231.

2. Shi YS, Fang W, Zhao XY, Li HX, Liu S. A Comparative Study on the Efficacy of Acupuncture Combined with Massage in the Treatment of Mild Carpal Tunnel Syndrome. *Chinese Journal of Integrated Traditional and Western Medicine* (2006) 26(6). Epub 20060930.

3. Luan YF, Zhou XC, Gong CX, Zhang K. 35 Cases of Carpal Tunnel Syndrome Treated by Acupuncture Plus He-Ne Laser. *Chinese Journal of Modern Chinese Medicine* (2008) 4(3). Epub 20090130.

4. Wang Y. Clinical Study on the Treatment of Mild to Moderate Carpal Tunnel Syndrome by Acupuncture through Tendon Junction [Master's dissertation]: Heilongjiang University of Traditional Chinese Medicine (2010).

5. Yang YZ. Clinical Study on Treatment of Carpal Tunnel Syndrome by Electroacupuncture and Shujin Washing Drug [Master's dissertation]: Guangzhou University of Traditional Chinese Medicine (2011).

6. Ding Q, Shen F. Effect of Yangchi Point Acupuncture on Carpal Tunnel Syndrome. *International Journal of Traditional Chinese Medicine* (2013) 35(2). Epub 20130628.

7. Maeda Y, Kettner N, Lee J, Kim J, Cina S, Malatesta C, et al. Acupuncture-Evoked Response in Somatosensory and Prefrontal Cortices Predicts Immediate Pain Reduction in Carpal Tunnel Syndrome. *Evid Based Complement Alternat Med* (2013) 2013:795906. Epub 2013/07/12. doi: 10.1155/2013/795906.

8. Duan QM, Zhang L, Wang R, Zhang L. Observation and Nursing Care of Electroacupuncture Combined with Physical Therapy for Carpal Tunnel Syndrome. *Journal of Nurses Training* (2014) 29(7). Epub 20141027.

9. Shou YQ, Jiang H, Chen WJ, Liu XJ, Wu XQ. Therapeutic Effect of Electroacupuncture Combined with Nerve Tendon Sliding Training on Mild to Moderate Carpal Tunnel Syndrome. *Chinese Journal of Physical Medicine and Rehabilitation* (2017) 39(1). Epub 20170605.

10. Wei XL. Clinical Observation of Electroacupuncture Combined with Shixuan Bloodletting in the Treatment of Mild to Moderate Carpal Tunnel Syndrome. *Hubei Journal of Traditional Chinese Medicine* (2017) 39(6). Epub 20171231.

11. Hu B. Effect of Electroacupuncture in Treating Carpal Tunnel Syndrome. *Journal of Frontiers of Medicine* (2018) 8(31):166-7. doi: 10.3969/j.issn.2095-1752.2018.31.129.

12. Yan LJ. Comparison of the Efficacy of Acupuncture Combined with Joint Loosening and Acupotomy in the Treatment of Carpal Tunnel Syndrome. *Chinese Journal of School Doctor* (2018) 32(6). Epub 20181230.

13. Kvist KB, Hilland R, Enehaug R, Schjelderup J, Lie SA, Halse AK. The Treatment Effect of Intramuscular Stimulation on Carpal Tunnel Syndrome: A Blinded Randomized Trial on 75 Patients. *J Bodyw Mov Ther* (2021) 27:522-8. Epub 2021/08/16. doi: 10.1016/j.jbmt.2021.03.020.

14. Wang XY. Clinical Effect of Hegu Acupuncture on Mild to Moderate Carpal Tunnel Syndrome [Mater's dissertation]: Tianjin University of Traditonal Chinese Medicine (2021).

**Ineligible outcomes (n =2)**

1 Salehi S, Hesami O, Esfehani MP, Khosravi S, Rashed A, Haghighatzadeh M, et al. The Effectiveness of Exercise Therapy and Dry Needling on Wrist Range of Motion, Pinch and Grip Force in Carpal Tunnel Syndrome: A Randomized Clinical Trial. Asian journal of sports medicine (2019) 10(4):1‐9. doi: 10.5812/asjsm.83927.

1. Feng, Y.P., and Shi, L. (2011). Fifty cases of carpal tunnel syndrome treated by acupuncture. Journal of Basic Chinese Medicine 17(06), 670-671.

**Unavailable data (n = 1)**

1. Weinstein A, Pan J, Richardson P. A Controlled Pilot Trial of Acupuncture for Carpal Tunnel Syndrome. Clin Acupunct Orient Med (2003) 4(1):48.

**Duplicates (n =2)**

1. Yang CP, Hsieh CL, Wang NH, Li TC, Hwang KL, Yu SC, et al. Acupuncture in Patients with Carpal Tunnel Syndrome a Randomized Controlled Trial. Clinical Journal of Pain (2009) 25(4):327-33. doi: 10.1097/AJP.0b013e318190511c.

2. Chung VC, Wong SY, Kung K, Zee CY, Leung WN, Chong KC, et al. Electroacupuncture and Wrist Splinting for Carpal Tunnel Syndrome: A Randomised Trial. Hong Kong medical journal = Xianggang yi xue za zhi (2017) 23 Suppl 2(3):28-31. Epub 2018/06/26.
