# Supplementary material for: Acupuncture for carpal tunnel syndrome: A systematic review and meta-analysis of randomized controlled trials
Source: Front Neurosci. 2023 Feb 23;17:1097455. doi: 10.3389/fnins.2023.1097455 (PMC9995832; doi:10.3389/fnins.2023.1097455)
Supplement: Supplementary file 3 [file Table_3.docx]

**The details of STRITA checklist of included studies**

|  | **1. Acupuncture rationale** | | | **2. Details of needling** | | | | | | | **3. Treatment regimen** | | **4. Cointerventions** | | **5. Practitioner background** | **6. Control or comparator interventions** | |
| --- | --- | --- | --- | --- | --- | --- | --- | --- | --- | --- | --- | --- | --- | --- | --- | --- | --- |
|  | **1a** | **1b** | **1c** | **2a** | **2b** | **2c** | **2d** | **2e** | **2f** | **2g** | **3a** | **3b** | **4a** | **4b** | **5** | **6a** | **6b** |
| Kumnerddee and Kaewtong (2010) | N | N | N | N | Y | Y | Y | Y | Y | Y | Y | Y | Y | N | Y | N | Y |
| Jin and Lang (2011) | N | N | N | N | N | N | Y | Y | N | Y | Y | N | N | N | N | N | Y |
| Li (2011) | N | N | N | N | Y | N | Y | Y | Y | Y | Y | Y | N | N | N | N | Y |
| Yang et al. (2011) | N | N | N | N | Y | Y | Y | Y | Y | Y | Y | Y | N | N | Y | N | Y |
| Yao et al. (2012) | Y | Y | N | Y | Y | N | Y | Y | Y | Y | Y | Y | N | N | Y | Y | Y |
| Ramin (2013) | N | N | N | N | Y | Y | Y | Y | Y | N | Y | Y | Y | N | N | N | Y |
| Xiang et al. (2014) | N | N | N | N | N | N | Y | Y | N | N | Y | Y | N | N | N | N | Y |
| Hadianfard et al. (2015) | N | Y | N | Y | Y | Y | Y | Y | Y | Y | Y | Y | N | N | Y | N | N |
| Chung et al. (2016) | Y | Y | N | N | Y | Y | Y | Y | Y | Y | Y | Y | N | N | Y | N | Y |
| Maeda et al. (2017) | N | Y | N | N | Y | Y | Y | Y | Y | Y | Y | Y | Y | N | Y | N | Y |
| Ural and Öztürk (2017) | N | Y | N | N | N | N | N | Y | Y | Y | Y | Y | N | N | N | N | Y |
| Xie et al. (2018) | N | N | N | N | Y | N | Y | Y | Y | Y | Y | Y | N | N | N | N | Y |
| Tezel et al. (2019) | N | Y | N | N | Y | N | N | Y | Y | Y | Y | Y | N | N | Y | N | N |
| Xiong et al. (2020) | N | N | N | N | Y | N | Y | Y | Y | Y | Y | Y | N | N | N | N | Y |
| Bahrami-Taghanaki et al. (2020) | Y | N | N | N | N | N | N | Y | N | N | Y | Y | N | N | Y | N | Y |
| Huang and Lin (2022) | N | N | N | N | N | Y | Y | Y | Y | Y | Y | Y | N | N | N | N | Y |
| 1a: Style of acupuncture (eg, TCM, Japanese, Korean, Western medical, Five Element, ear acupuncture, etc);  1b: Reasoning for treatment provided, based on historical context, literature sources, and consensus methods, with references where appropriate;  1c: Extent to which treatment was varied;  2a: Number of needle insertions per subject per session (mean and range where relevant);  2b: Names (or location if no standard name) of points used (uni/bilateral);  2c: Depth of insertion, based on a specified unit of measurement, or on a particular tissue level;  2d: Response sought (eg, de qi or muscle twitch response);  2e: Needle stimulation (eg, manual, electrical);  2f: Needle retention time;  2g: Needle type (diameter, length, and manufacturer or material);  3a: Number of treatment sessions;  3b: Frequency and duration of treatment sessions;  4a: Details of other interventions administered to the acupuncture group (eg, moxibustion, cupping, herbs, exercises, lifestyle advice);  4b: Setting and context of treatment, including instructions to practitioners, and information and explanations to patients;  5: Description of participating acupuncturists (qualification or professional affiliation, years in acupuncture practice, other relevant experience);  6a: Rationale for the control or comparator in the context of the research question, with sources that justify this choice;  6b: Precise description of the control or comparator. If sham acupuncture or any other type of acupuncture-like control is used, provide details as for Items 1 to 3 above. | | | | | | | | | | | | | | | | | |
