# Supplementary material for: Acupuncture for carpal tunnel syndrome: A systematic review and meta-analysis of randomized controlled trials
Source: Front Neurosci. 2023 Feb 23;17:1097455. doi: 10.3389/fnins.2023.1097455 (PMC9995832; doi:10.3389/fnins.2023.1097455)
Supplement: Supplementary file 4 [file Table_4.docx]

| **Acupuncture compared to sham acupuncture for carpal tunnel syndrome** | | | | | | |
| --- | --- | --- | --- | --- | --- | --- |
| **Patient or population:** Patients with carpal tunnel syndrome **Settings:**  **Intervention:** Acupuncture  **Comparison:** Sham acupuncture | | | | | | |
| **Outcomes** | **Illustrative comparative risks* (95% CI)** | | **Relative effect (95% CI)** | **No of Participants (studies)** | **Quality of the evidence (GRADE)** | **Comments** |
|  | Assumed risk | Corresponding risk |  |  |  |  |
|  | **Sham acupuncture** | **Acupuncture** |  |  |  |  |
| **Functional status** CTQ-FSS |  | The mean functional status in the intervention groups was **8 lower** (24.44 lower to 8.44 higher) |  | 43 (1 study) | ⊕⊕⊝⊝ **low** |  |
| **Symptom severity** CTQ-SSS |  | The mean symptom severity in the intervention groups was **4.30 lower** (17.90 lower to 9.30 higher) |  | 43 (1 study) | ⊕⊝⊝⊝ **very low** |  |
| *The basis for the **assumed risk** (e.g. the median control group risk across studies) is provided in footnotes. The **corresponding risk** (and its 95% confidence interval) is based on the assumed risk in the comparison group and the **relative effect** of the intervention (and its 95% CI). **CI:** Confidence interval; **CTQ-SSS**: the Boston Carpal Tunnel Questionnaire’s symptom severity scale; **CTQ-FSS**: CTQ’s functional status scale. | | | | | | |
| GRADE Working Group grades of evidence **High quality:** Further research is very unlikely to change our confidence in the estimate of effect.  **Moderate quality:** Further research is likely to have an important impact on our confidence in the estimate of effect and may change the estimate. **Low quality:** Further research is very likely to have an important impact on our confidence in the estimate of effect and is likely to change the estimate. **Very low quality:** We are very uncertain about the estimate. | | | | | | |

| **Acupuncture compared to night splints for carpal tunnel syndrome** | | | | | | |
| --- | --- | --- | --- | --- | --- | --- |
| **Patient or population:** Patients with carpal tunnel syndrome **Settings:**  **Intervention:** Acupuncture **Comparison:** Night splints | | | | | | |
| **Outcomes** | **Illustrative comparative risks* (95% CI)** | | **Relative effect (95% CI)** | **No of Participants (studies)** | **Quality of the evidence (GRADE)** | **Comments** |
|  | Assumed risk | Corresponding risk |  |  |  |  |
|  | **Night splints** | **Acupuncture** |  |  |  |  |
| **Symptom severity** CTQ-SSS |  | The mean symptom severity in the intervention groups was **0.09 lower** (0.32 lower to 0.14 higher) |  | 60 (1 study) | ⊕⊕⊝⊝ **low** |  |
| **Functional status** CTQ-FSS |  | The mean functional status in the intervention groups was **0.04 lower** (0.26 lower to 0.18 higher) |  | 60 (1 study) | ⊕⊝⊝⊝ **very low** |  |
| **VAS** |  | The mean VAS in the intervention groups was **9.63 lower** (19.27 lower to 0.01 higher) |  | 60 (1 study) | ⊕⊝⊝⊝ **very low** |  |
| *The basis for the **assumed risk** (e.g. the median control group risk across studies) is provided in footnotes. The **corresponding risk** (and its 95% confidence interval) is based on the assumed risk in the comparison group and the **relative effect** of the intervention (and its 95% CI).  **CI:** Confidence interval; **CTQ-SSS**: the Boston Carpal Tunnel Questionnaire’s symptom severity scale; **CTQ-FSS**: CTQ’s functional status scale; **VAS**: the visual analog scale. | | | | | | |
| GRADE Working Group grades of evidence **High quality:** Further research is very unlikely to change our confidence in the estimate of effect.  **Moderate quality:** Further research is likely to have an important impact on our confidence in the estimate of effect and may change the estimate. **Low quality:** Further research is very likely to have an important impact on our confidence in the estimate of effect and is likely to change the estimate. **Very low quality:** We are very uncertain about the estimate. | | | | | | |

| **Acupuncture compared to medicine for carpal tunnel syndrome** | | | | | | |
| --- | --- | --- | --- | --- | --- | --- |
| **Patient or population:** Patients with carpal tunnel syndrome **Settings:**  **Intervention:** Acupuncture **Comparison:** Medicine | | | | | | |
| **Outcomes** | **Illustrative comparative risks* (95% CI)** | | **Relative effect (95% CI)** | **No of Participants (studies)** | **Quality of the evidence (GRADE)** | **Comments** |
|  | Assumed risk | Corresponding risk |  |  |  |  |
|  | **Medicine** | **Acupuncture** |  |  |  |  |
| **Symptom severity** GSS |  | The mean symptom severity in the intervention groups was **0.6 lower** (2.12 lower to 0.92 higher) |  | 77 (1 study) | ⊕⊕⊝⊝ **low** |  |
| **CAMP** |  | The mean CAMP in the intervention groups was **1.02 lower** (2.02 lower to 0.03 lower) |  | 179 (3 studies) | ⊕⊝⊝⊝ **very low** |  |
| **DML** |  | The mean DML in the intervention groups was **0.31 lower** (0.96 lower to 0.34 higher) |  | 179 (3 studies) | ⊕⊝⊝⊝ **very low** |  |
| **DSL** |  | The mean DSL in the intervention groups was **0.05 lower** (0.78 lower to 0.69 higher) |  | 129 (2 studies) | ⊕⊝⊝⊝ **very low** |  |
| **MNCV** |  | The mean MNCV in the intervention groups was **3.57 lower** (13.79 lower to 6.65 higher) |  | 129 (2 studies) | ⊕⊝⊝⊝ **very low** |  |
| **SNCV** |  | The mean SNCV in the intervention groups was **1.12** **lower** (6.39 lower to 4.14 higher) |  | 179 (3 studies) | ⊕⊝⊝⊝ **very low** |  |
| **SNAP** |  | The mean SNAP in the intervention groups was **3.14 lower** (6.84 lower to 0.56 higher) |  | 129 (2 studies) | ⊕⊝⊝⊝ **very low** |  |
| **Responder rate** | **Study population** | | **RR 1.1**  (0.86 to 1.4) | 50 (1 study) | ⊕⊝⊝⊝ **very low** |  |
|  | **800 per 1000** | **880 per 1000** (688 to 1000) |  |  |  |  |
|  | **Moderate** | |  |  |  |  |
|  | **800 per 1000** | **880 per 1000** (688 to 1000) |  |  |  |  |
| *The basis for the **assumed risk** (e.g. the median control group risk across studies) is provided in footnotes. The **corresponding risk** (and its 95% confidence interval) is based on the assumed risk in the comparison group and the **relative effect** of the intervention (and its 95% CI).  **CI:** Confidence interval; **RR:** Risk ratio; **GSS**: the global symptoms score; **CAMP:** compound muscle action potential; **DML**: distal motor latency; **DSL**: distal sensory latency; **MNCV**: motor nerve conduction velocity; **SNCV**: sensory nerve conduction velocity; **SNAP**: sensory nerve action potential. | | | | | | |
| GRADE Working Group grades of evidence **High quality:** Further research is very unlikely to change our confidence in the estimate of effect.  **Moderate quality:** Further research is likely to have an important impact on our confidence in the estimate of effect and may change the estimate. **Low quality:** Further research is very likely to have an important impact on our confidence in the estimate of effect and is likely to change the estimate. **Very low quality:** We are very uncertain about the estimate. | | | | | | |

| **Acupuncture plus night splints compared to sham acupuncture plus night splints for carpal tunnel syndrome** | | | | | | |
| --- | --- | --- | --- | --- | --- | --- |
| **Patient or population:** Patients with carpal tunnel syndrome **Settings:**  **Intervention:** Acupuncture plus night splints **Comparison:** Sham acupuncture plus night splints | | | | | | |
| **Outcomes** | **Illustrative comparative risks* (95% CI)** | | **Relative effect (95% CI)** | **No of Participants (studies)** | **Quality of the evidence (GRADE)** | **Comments** |
|  | Assumed risk | Corresponding risk |  |  |  |  |
|  | **Sham acupuncture night splints** | **Acupuncture plus night splints** |  |  |  |  |
| **Symptom severity** CTQ-SSS |  | The mean symptom severity in the intervention groups was **0.20 lower** (0.75 lower to 0.35 higher) |  | 41 (1 study) | ⊕⊕⊝⊝ **low** |  |
| **Functional status** CTQ-FSS |  | The mean functional status in the intervention groups was **0.00 lower** (0.64 lower to 0.64 higher) |  | 41 (1 study) | ⊕⊕⊝⊝ **low** |  |
| *The basis for the **assumed risk** (e.g. the median control group risk across studies) is provided in footnotes. The **corresponding risk** (and its 95% confidence interval) is based on the assumed risk in the comparison group and the **relative effect** of the intervention (and its 95% CI).  **CI:** Confidence interval; **CTQ-SSS**: the Boston Carpal Tunnel Questionnaire’s symptom severity scale; **CTQ-FSS**: CTQ’s functional status scale. | | | | | | |
| GRADE Working Group grades of evidence **High quality:** Further research is very unlikely to change our confidence in the estimate of effect.  **Moderate quality:** Further research is likely to have an important impact on our confidence in the estimate of effect and may change the estimate. **Low quality:** Further research is very likely to have an important impact on our confidence in the estimate of effect and is likely to change the estimate. **Very low quality:** We are very uncertain about the estimate. | | | | | | |

| **Acupuncture plus night splints compared to medicine plus night splints for carpal tunnel syndrome** | | | | | | |
| --- | --- | --- | --- | --- | --- | --- |
| **Patient or population:** Patients with carpal tunnel syndrome **Settings:**  **Intervention:** Acupuncture plus night splints **Comparison:** Medicine plus night splints | | | | | | |
| **Outcomes** | **Illustrative comparative risks* (95% CI)** | | **Relative effect (95% CI)** | **No of Participants (studies)** | **Quality of the evidence (GRADE)** | **Comments** |
|  | Assumed risk | Corresponding risk |  |  |  |  |
|  | **Medicine+Night splints** | **Acupuncture+Night splints** |  |  |  |  |
| **Symptom severity** CTQ-SSS/GSS |  | The mean symptom severity in the intervention groups was **1.15 standard deviations lower** (1.58 to 0.72 lower) |  | 99 (2 studies) | ⊕⊝⊝⊝ **very low** |  |
| **Functional status** CTQ-FSS |  | The mean functional status in the intervention groups was **1.84 lower** (2.66 to 1.02 lower) |  | 50 (1 study) | ⊕⊕⊝⊝ **low** |  |
| **MNCV** |  | The mean MNCV in the intervention groups was **1.76 higher** (0.68 to 2.84 higher) |  | 50 (1 study) | ⊕⊕⊝⊝ **low** |  |
| **DML** |  | The mean DML in the intervention groups was **0.2 lower** (0.43 lower to 0.03 higher) |  | 50 (1 study) | ⊕⊕⊝⊝ **low** |  |
| **DSL** |  | The mean DSL in the intervention groups was **0.26 lower** (0.37 to 0.15 lower) |  | 50 (1 study) | ⊕⊕⊝⊝ **low** |  |
| **VAS** |  | The mean VAS in the intervention groups was **0.84 lower** (1.25 to 0.43 lower) |  | 50 (1 study) | ⊕⊕⊝⊝ **low**^2,3,4^ |  |
| *The basis for the **assumed risk** (e.g. the median control group risk across studies) is provided in footnotes. The **corresponding risk** (and its 95% confidence interval) is based on the assumed risk in the comparison group and the **relative effect** of the intervention (and its 95% CI).  **CI:** Confidence interval; **CTQ-SSS**: the Boston Carpal Tunnel Questionnaire’s symptom severity scale; **CTQ-FSS**: CTQ’s functional status scale; **MNCV**: motor nerve conduction velocity; **DML**: distal  motor latency; **DSL**: distal sensory latency; **VAS**: the visual analog scale. | | | | | | |
| GRADE Working Group grades of evidence **High quality:** Further research is very unlikely to change our confidence in the estimate of effect.  **Moderate quality:** Further research is likely to have an important impact on our confidence in the estimate of effect and may change the estimate. **Low quality:** Further research is very likely to have an important impact on our confidence in the estimate of effect and is likely to change the estimate. **Very low quality:** We are very uncertain about the estimate. | | | | | | |

| **Acupuncture plus night splints compared to night splints for carpal tunnel syndrome** | | | | | | |
| --- | --- | --- | --- | --- | --- | --- |
| **Patient or population:** Patients with carpal tunnel syndrome **Settings:**  **Intervention:** Acupuncture plus night splints **Comparison:** Night splints | | | | | | |
| **Outcomes** | **Illustrative comparative risks* (95% CI)** | | **Relative effect (95% CI)** | **No of Participants (studies)** | **Quality of the evidence (GRADE)** | **Comments** |
|  | Assumed risk | Corresponding risk |  |  |  |  |
|  | **Night splints** | **Acupuncture plus night splints** |  |  |  |  |
| **Symptom severity**  CTQ-SSS |  | The mean symptom severity in the intervention groups was **0.13** **standard deviations lower** (0.59 lower to 0.32 higher) |  | 225 (2 studies) | ⊕⊝⊝⊝ **very low** |  |
| **Functional status** CTQ-FSS |  | The mean functional status in the intervention groups was **0.2 standard deviations lower** (0.87 lower to 0.46 higher) |  | 225 (2 studies) | ⊕⊝⊝⊝ **very low** |  |
| **Functional status** DASH (change) |  | The mean functional status in the intervention groups was **0.40 standard deviations lower** (0.68 to 0.13 lower) |  | 208 (2 studies) | ⊕⊝⊝⊝ **very low** |  |
| **VAS** |  | The mean VAS in the intervention groups was **1.65 lower** (3.05 to 0.26 lower) |  | 252 (3 studies) | ⊕⊕⊝⊝ **low** |  |
| **CMAP** |  | The mean CMAP in the intervention groups was **1.31 higher** (1.04 lower to 3.66 higher) |  | 71 (2 studies) | ⊕⊝⊝⊝ **very low** |  |
| **MNCV** |  | The mean MNCV in the intervention groups was **1.81 higher** (0.55 lower to 4.18 higher) |  | 71 (2 studies) | ⊕⊝⊝⊝ **very low** |  |
| **DML** |  | The mean DML in the intervention groups was **0.05 higher** (0.33 lower to 0.43 higher) |  | 71 (2 studies) | ⊕⊝⊝⊝ **very low** |  |
| **SNAP** |  | The mean SNAP in the intervention groups was **3.2 higher** (0.73 lower to 7.13 higher) |  | 27 (1 study) | ⊕⊝⊝⊝ **very low** |  |
| **SNCV** |  | The mean SNCV in the intervention groups was **0.24 higher** (2.2 lower to 2.67 higher) |  | 71 (2 studies) | ⊕⊝⊝⊝ **very low** |  |
| *The basis for the **assumed risk** (e.g. the median control group risk across studies) is provided in footnotes. The **corresponding risk** (and its 95% confidence interval) is based on the assumed risk in the comparison group and the **relative effect** of the intervention (and its 95% CI).  **CI:** Confidence interval; **CTQ-SSS**: the Boston Carpal Tunnel Questionnaire’s symptom severity scale; **CTQ-FSS**: CTQ’s functional status scale; **DASH**: the disabilities of the arm, shoulder, and hand  questionnaire; **VAS**: the visual analog scale; **CAMP**: compound muscle action potential; **MNCV**: motor nerve conduction velocity; **DML**: distal motor latency; **SMAP**: sensory nerve action potential; **SNCV**: sensory nerve conduction velocity; **DSL**: distal sensory latency. | | | | | | |
| GRADE Working Group grades of evidence **High quality:** Further research is very unlikely to change our confidence in the estimate of effect.  **Moderate quality:** Further research is likely to have an important impact on our confidence in the estimate of effect and may change the estimate. **Low quality:** Further research is very likely to have an important impact on our confidence in the estimate of effect and is likely to change the estimate. **Very low quality:** We are very uncertain about the estimate. | | | | | | |

| **Acupuncture plus medicine compared to medicine for carpal tunnel syndrome** | | | | | | |
| --- | --- | --- | --- | --- | --- | --- |
| **Patient or population:** Patients with carpal tunnel syndrome **Settings:**  **Intervention:** Acupuncture plus medicine **Comparison:** Medicine | | | | | | |
| **Outcomes** | **Illustrative comparative risks* (95% CI)** | | **Relative effect (95% CI)** | **No of Participants (studies)** | **Quality of the evidence (GRADE)** | **Comments** |
|  | Assumed risk | Corresponding risk |  |  |  |  |
|  | **Medicine** | **Acupuncture plus medicine** |  |  |  |  |
| **Symptom severity** CTQ-SSS/GSS |  | The mean symptom severity in the intervention groups was **1.17 standard deviations lower** (2.31 to 0.03 lower) |  | 226 (3 studies) | ⊕⊝⊝⊝ **very low** |  |
| **Functional status** CTQ-FSS |  | The mean functional status in the intervention groups was **2.17 lower** (6.45 lower to 2.1 higher) |  | 166 (2 studies) | ⊕⊝⊝⊝ **very low** |  |
| **VAS** |  | The mean VAS in the intervention groups was **1.08 lower** (1.82 to 0.33 lower) |  | 60 (1 study) | ⊕⊝⊝⊝ **very low** |  |
| **SNAP** |  | The mean SNAP in the intervention groups was **2.53 higher** (1.63 to 3.44 higher) |  | 226 (3 studies) | ⊕⊕⊝⊝ **low** |  |
| **CMAP** |  | The mean CMAP in the intervention groups was **2.30 higher** (0.84 to 3.77 higher) |  | 226 (3 studies) | ⊕⊝⊝⊝ **very low** |  |
| **DML** |  | The mean DML in the intervention groups was **0.47 lower** (0.66 to 0.28 lower) |  | 226 (3 studies) | ⊕⊝⊝⊝ **very low** |  |
| **SNCV** |  | The mean SNCV in the intervention groups was **4.02 higher** (2.44 to 5.59 higher) |  | 226 (3 studies) | ⊕⊝⊝⊝ **very low** |  |
| **Responder rate** | **Study population** | | **RR 1.2**  (1.03 to 1.39) | 86 (1 study) | ⊕⊝⊝⊝ **very low** |  |
|  | **814 per 1000** | **977 per 1000** (838 to 1000) |  |  |  |  |
|  | **Moderate** | |  |  |  |  |
|  | **807 per 1000** | **968 per 1000** (831 to 1000) |  |  |  |  |
| *The basis for the **assumed risk** (e.g. the median control group risk across studies) is provided in footnotes. The **corresponding risk** (and its 95% confidence interval) is based on the assumed risk in the comparison group and the **relative effect** of the intervention (and its 95% CI).  **CI:** Confidence interval; **RR:** Risk ratio; **CTQ-SSS**: the Boston Carpal Tunnel Questionnaire’s symptom severity scale; **CTQ-FSS**: CTQ’s functional status scale; **GSS**: the global symptoms score; **VAS**: the visual analog scale; **CAMP**: compound muscle action potential; **SMAP**: sensory nerve action potential; **DML**: distal motor latency; **SNCV**: sensory nerve conduction velocity. | | | | | | |
| GRADE Working Group grades of evidence **High quality:** Further research is very unlikely to change our confidence in the estimate of effect.  **Moderate quality:** Further research is likely to have an important impact on our confidence in the estimate of effect and may change the estimate. **Low quality:** Further research is very likely to have an important impact on our confidence in the estimate of effect and is likely to change the estimate. **Very low quality:** We are very uncertain about the estimate. | | | | | | |

| **Acupuncture plus medicine and night splints compared to medicine plus night splints for carpal tunnel syndrome** | | | | | | |
| --- | --- | --- | --- | --- | --- | --- |
| **Patient or population:** Patients with carpal tunnel syndrome **Settings:**  **Intervention:** Acupuncture plus medicine plus night splints **Comparison:** Medicine plus night splints | | | | | | |
| **Outcomes** | **Illustrative comparative risks* (95% CI)** | | **Relative effect (95% CI)** | **No of Participants (studies)** | **Quality of the evidence (GRADE)** | **Comments** |
|  | Assumed risk | Corresponding risk |  |  |  |  |
|  | **Medicine plus night splints** | **Acupuncture plus medicine and night splints** |  |  |  |  |
| **Symptom severity** CTQ-SSS |  | The mean symptom severity in the intervention groups was **5.67 lower** (7.82 to 3.52 lower) |  | 50 (1 study) | ⊕⊝⊝⊝ **very low** |  |
| **Functional status** CTQ-FSS |  | The mean functional status in the intervention groups was **0.70 lower** (1.41 lower to 0.01 higher) |  | 50 (1 study) | ⊕⊝⊝⊝ **very low** |  |
| **VAS** |  | The mean VAS in the intervention groups was **0.64 lower** (1.08 to 0.2 lower) |  | 50 (1 study) | ⊕⊝⊝⊝ **very low** |  |
| **DML** |  | The mean DML in the intervention groups was **0.22 lower** (0.48 lower to 0.04 higher) |  | 50 (1 study) | ⊕⊝⊝⊝ **very low** |  |
| **DSL** |  | The mean DSL in the intervention groups was **0.53 lower** (0.75 to 0.31 lower) |  | 50 (1 study) | ⊕⊝⊝⊝ **very low** |  |
| *The basis for the **assumed risk** (e.g. the median control group risk across studies) is provided in footnotes. The **corresponding risk** (and its 95% confidence interval) is based on the assumed risk in the comparison group and the **relative effect** of the intervention (and its 95% CI).  **CI:** Confidence interval; **CTQ-SSS**: the Boston Carpal Tunnel Questionnaire’s symptom severity scale; **CTQ-FSS**: CTQ’s functional status scale; **VAS**: the visual analog scale; **DML**: distal motor latency; **DSL**: distal sensory latency. | | | | | | |
| GRADE Working Group grades of evidence **High quality:** Further research is very unlikely to change our confidence in the estimate of effect.  **Moderate quality:** Further research is likely to have an important impact on our confidence in the estimate of effect and may change the estimate. **Low quality:** Further research is very likely to have an important impact on our confidence in the estimate of effect and is likely to change the estimate. **Very low quality:** We are very uncertain about the estimate. | | | | | | |

| **Acupuncture plus ultrashort wave therapy compared to ultrashort wave therapy for carpal tunnel syndrome** | | | | | | |
| --- | --- | --- | --- | --- | --- | --- |
| **Patient or population:** Patients with carpal tunnel syndrome **Settings:**  **Intervention:** Acupuncture plus ultrashort wave therapy  **Comparison:** Ultrashort wave therapy | | | | | | |
| **Outcomes** | **Illustrative comparative risks* (95% CI)** | | **Relative effect (95% CI)** | **No of Participants (studies)** | **Quality of the evidence (GRADE)** | **Comments** |
|  | Assumed risk | Corresponding risk |  |  |  |  |
|  | **Ultrashort wave therapy** | **Acupuncture plus ltrashort wave therapy** |  |  |  |  |
| **Symptom severity** CTQ-SSS |  | The mean symptom severity in the intervention groups was **0.86 lower** (1.27 to 0.45 lower) |  | 48 (1 study) | ⊕⊝⊝⊝ **very low** |  |
| **Responder rate** | **Study population** | | **RR 1.1**  (0.89 to 1.36) | 48 (1 study) | ⊕⊝⊝⊝ **very low** |  |
|  | **833 per 1000** | **917 per 1000** (742 to 1000) |  |  |  |  |
|  | **Moderate** | |  |  |  |  |
|  | **833 per 1000** | **916 per 1000** (741 to 1000) |  |  |  |  |
| *The basis for the **assumed risk** (e.g. the median control group risk across studies) is provided in footnotes. The **corresponding risk** (and its 95% confidence interval) is based on the assumed risk in the comparison group and the **relative effect** of the intervention (and its 95% CI).  **CI:** Confidence interval; **RR:** Risk ratio; **CTQ-SSS**: the Boston Carpal Tunnel Questionnaire’s symptom severity scale. | | | | | | |
| GRADE Working Group grades of evidence **High quality:** Further research is very unlikely to change our confidence in the estimate of effect.  **Moderate quality:** Further research is likely to have an important impact on our confidence in the estimate of effect and may change the estimate. **Low quality:** Further research is very likely to have an important impact on our confidence in the estimate of effect and is likely to change the estimate. **Very low quality:** We are very uncertain about the estimate. | | | | | | |
